# Obesity mediates the association of outdoor artificial light at night with type 2 diabetes mellitus

**DOI:** 10.1016/j.isci.2026.115036

**Published:** 2026-02-26

**Authors:** Xiaotian Liu, Zhongao Ding, Yinghao Yuchi, Ruiying Li, Wei Liao, Xiaokang Dong, Wenqian Huo, Jian Hou, Hualiang Lin, Xin Liu, Kai Zhang, Chongjian Wang

**Affiliations:** 1 Department of Epidemiology and Biostatistics, College of Public Health, Zhengzhou University, Zhengzhou, Henan, P.R. China; 2 NHC Key Laboratory of Prevention and Treatment of Cerebrovascular Diseases, Zhengzhou, Henan, P.R. China; 3 Department of Epidemiology, School of Public Health, Sun Yat-sen University, Guangzhou, Guangdong, P.R. China; 4 School of Public Health and Emergency Management, Southern University of Science and Technology, Shenzhen, Guangdong, P.R. China; 5 Department of Environmental Health Sciences, College of Integrated Health Sciences, University at Albany, Rensselaer, NY, USA

**Keywords:** Health sciences

## Abstract

This study aims to explore the association of outdoor ALAN exposure with T2DM and the mediating effects of obesity in Chinese rural adults. A total of 38,108 participants were recruited from the Henan Rural Cohort. ALAN was positively associated with T2DM and FPG, and negatively associated with insulin, HOMA-IR, as well as HOMA-β, and the effect sizes of per-quartile increase in ALAN were 1.24(1.19, 1.28), 0.14(0.13, 0.16), −1.19(-1.24, −1.14), −0.22(-0.25, −0.20), and −27.30(-29.11, −25.48), respectively. The elderly, men, current smokers, individuals with lower education or income, or physical inactivity were more vulnerable to ALAN-related adverse health effects. The associations of ALAN with T2DM and glucose metabolism were mediated by WC, BMI, WHtR, WHR, VFI, and BFP, with estimated mediation proportions of 36.41%, 25.14%, 33.98%, 22.33%, 24.39%, and 25.85%, respectively, for T2DM. These findings emphasize the need for targeted public health interventions for reducing ALAN-related adverse health effects in rural areas.

## Highlights

• ALAN was positively associated with T2DM and impaired glucose metabolism in rural areas
• High-risk individuals with T2DM might benefit from controlling outdoor ALAN exposure
• Obese indices appear to be key mediators of these associations, regardless of types
• These findings provide evidence for promoting public health by reducing ALAN exposure

## Graphical abstract

**Figure:**
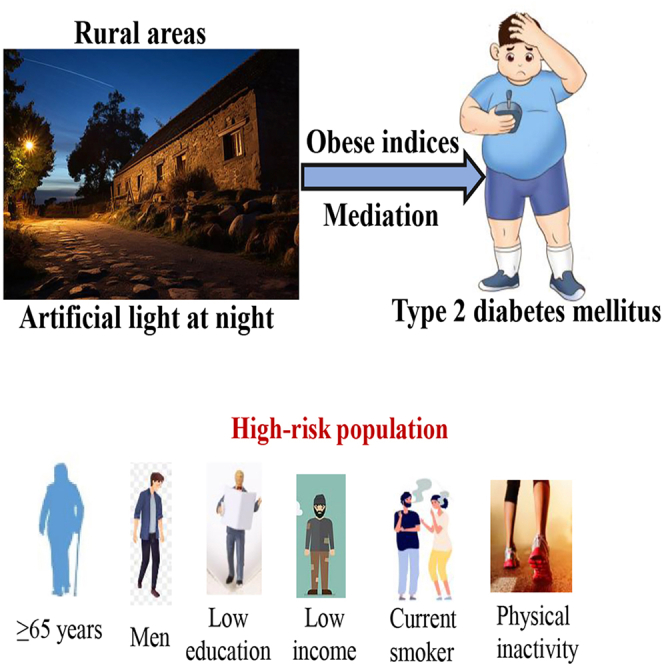

## Introduction

Diabetes has become one of the fastest-growing global health emergencies of the 21st century. It is estimated that 588.7 million adults aged 20–79 were living with diabetes in 2024 estimated by the International Diabetes Federation (IDF), which means that one in nine adults now living with this condition.^1^ As the main type of diabetes, type 2 diabetes mellitus (T2DM) affects individuals across various life stages^1^^,^^2^^,^^3^ A growing number of studies pointed to the effect of environmental risk factors on the development of T2DM.^4^^,^^5^ Among them, artificial light at night (ALAN), a prevalent form of light pollution, has received increasing attention in the environmental health field, such as obesity and metabolic diseases.^6^^,^^7^^,^^8^^,^^9^

Over the course of human evolution, circadian rhythms are important risk factors for maintaining homeostatic balance.^10^ With the extended use of artificial light, such as outdoor/indoor light, mobile phone, television, computers, among others, the population is exposed to excessive ALAN, leading to an increase in photoperiod and disrupting the circadian rhythm.^11^^,^^12^ After long-term exposure to ALAN for four weeks, impaired glucose tolerance and higher body weight were observed in mice.^13^ Epidemiological studies have also highlighted the connection between outdoor ALAN and diabetes.^14^^,^^15^ The China Noncommunicable Disease Surveillance Study indicated that a higher intensity of outdoor ALAN increased the prevalence of diabetes.^16^ In Japan, a study reported that ALAN exposure increased the incidence risk of diabetes in the elderly population.^17^ Notably, previous studies have concentrated on the adverse effects of ALAN exposure in urban areas because ALAN exposure was often primarily recognized as an urban issue.^15^^,^^18^ However, nearly 46% of the global population resides in non-urban areas, and rural areas contributed 40% of the total ALAN emissions.^19^ Nevertheless, few studies have investigated ALAN’s specific influence on rural residents while outdoor ALAN exposure is gradually increasing with the development of society.

Obesity has become the leading risk factor for T2DM.^20^^,^^21^ While animal experiments have hinted at the mediating role of body weight in the association between ALAN exposure and T2DM,^22^ evidence from the population study was limited to verifying the association. Furthermore, due to different measurement emphasis and predicting the risk of disease, many anthropometric indicators have been put forward to define obesity, such as waist circumference (WC), body mass index (BMI), waist-to-height ratio (WHtR), waist-to-hip ratio (WHR), visceral fat index (VFI), and body fat percentage (BFP).^23^^,^^24^^,^^25^^,^^26^^,^^27^^,^^28^ WC, WHtR, and WHR were used to assess abdominal obesity, but WC and WHtR were more advantageous to predict cardiovascular disease (CVD) risk compared with WHR.^23^^,^^24^^,^^25^^,^^26^ BMI was applied to assess general obesity.^25^ VFI highlighted the visceral adipose tissue, while BFP emphasized the proportion of body fat in weight.^27^^,^^28^ However, there was controversial that which was the best anthropometric indicator to define obesity.

Therefore, this study aims to examine the associations of outdoor ALAN with T2DM, fasting plasma glucose (FPG), fasting insulin, insulin resistance index (HOMA-IR), and β-cell function index (HOMA-β) in a Chinese rural population. Moreover, the potential modification effects of demographic factors and lifestyle were also assessed. Simultaneously, the potential mediation roles of different obese anthropometric indices (WC, BMI, WHtR, WHR, VFI, and BFP) in the associations were explored. These findings will help to provide a scientific basis for developing T2DM management policies related to outdoor ALAN in limited-resource areas.

## Results

### Characteristics of the participants

The characteristics of the enrolled participants are shown in Table 1. Among the 38,105 participants, 39.16% were men, and the mean age was 55.53 years. Nearly half of the residents have not attended school or have only received a primary school education. Additionally, more than two-thirds (68.44%) of the participants got less than 1000 RMB per month. Participants with T2DM were more likely to be older, women, have lower levels of education, income, and physical activity, inadequate vegetable and fruit intake, higher WC, BMI, WHtR, WHR, VFI, and BFP (*P* < 0.05). The mean of outdoor ALAN exposure level in participants with T2DM was higher than that in the participants without T2DM (1.50 nW/cm^2^/sr vs. 1.28 nW/cm^2^/sr).

**Table 1:** Characteristics of the participants according to T2DM status

Variable	T2DM (*n* = 3544)	Non-T2DM (*n* = 34561)	*χ*^*2*^*/t*	*P*
Age (years, mean ± SD)	60.36 ± 9.25	55.04 ± 12.36	24.947	<0.001
Men, n (%)	1329 (37.50)	13593 (39.33)	4.520	0.033
Marital status, n (%)	–	–	5.493	0.019
Married/cohabiting	3141 (88.63)	31064 (89.88)	–	–
Widowed/single/divorced/separated	403 (11.37)	3497 (10.12)	–	–
Education, n (%)	–	–	178.359	<0.001
Primary school or below	1967 (55.50)	15133 (43.79)	–	–
Junior high school or above	1577 (44.50)	19428 (56.21)	–	–
Per capita monthly income, n (%)	–	–	26.180	<0.001
<500 RMB	1388 (39.16)	12127 (35.09)	–	–
500-999RMB	1140 (32.17)	11424 (33.05)	–	–
≥1000RMB	1016 (28.67)	11010 (31.86)	–	–
Smoking, n (%)	–	–	64.454	<0.001
Never	2686 (75.79)	25169 (72.82)	–	–
Ever	351 (9.90)	2705 (7.83)	–	–
Current	507 (14.31)	6687 (19.35)	–	–
Drinking, n (%)	–	–	46.072	<0.001
Never	2807 (79.20)	26721 (77.32)	–	–
Ever	219 (6.18)	1536 (4.44)	–	–
Current	518 (14.62)	6304 (18.24)	–	–
More vegetable and fruit intake, n (%)	1255 (35.41)	14693 (42.51)	66.629	<0.001
High-fat diet, n (%)	579 (16.34)	6767 (19.58)	21.716	<0.001
Physical activity, n (%)	–	–	92.495	<0.001
Low	1389 (39.19)	10897 (31.53)	–	–
Moderate	1264 (35.67)	13195 (38.18)	–	–
High	891 (25.14)	10469 (30.29)	–	–
Family history of diabetes, n (%)	349 (9.85)	1235 (3.57)	317.604	<0.001
WC (cm, mean ± SD)	89.40 ± 10.09	83.61 ± 10.27	31.909	<0.001
BMI (kg/m^2^, mean ± SD)	26.24 ± 3.64	24.73 ± 3.52	24.114	<0.001
WHtR (mean ± SD)	0.56 ± 0.06	0.52 ± 0.06	34.161	<0.001
WHR (mean ± SD)	0.93 ± 0.07	0.88 ± 0.07	37.221	<0.001
VFI (mean ± SD)	11.49 ± 4.93	9.27 ± 4.50	27.545	<0.001
BFP (%, mean ± SD)	32.55 ± 6.20	29.88 ± 6.64	22.746	<0.001
FPG (mmol/L, mean ± SD)	8.92 ± 2.85	5.19 ± 0.57	206.496	<0.001
Insulin (mIU/L, mean ± SD)	12.59 ± 7.21	10.72 ± 5.06	20.010	<0.001
HOMA-IR (mean ± SD)	5.00 ± 3.41	2.48 ± 1.27	43.511	<0.001
HOMA-β (mean ± SD)	58.83 ± 64.13	145.99 ± 195.53	−57.894	<0.001
ALAN (nW/cm^2^/sr, mean ± SD)	1.50 ± 1.81	1.28 ± 1.86	6.710	<0.001

SD, standard deviation; WC: waist circumference; BMI, body mass index; WHtR: waist-to-height ratio; WHR: waist-to-hip ratio; VFI: visceral fat index; BFP: body fat percentage; FPG, fasting plasma glucose; HOMA-IR: insulin resistance index; HOMA-β: β-cell function index; ALAN: artificial light at night.

### Associations of outdoor artificial light at night exposure with type 2 diabetes mellitus

To examine the associations of outdoor ALAN exposure with T2DM more accurately, quartile, per-quartile, and a continuous variable of ALAN exposure were employed in the models. The results are shown in Table 2. In Model 3 adjusting for potential confounders including age, gender, education status, marital status, per capita monthly income, smoking and drinking status, more vegetable and fruit intake, high-fat diet, physical activity, and family history of diabetes, the odds ratio (*OR*) and 95% confidence interval (*CI*) for T2DM risk in the highest quartile, per-quartile increase and continuous variable of ALAN exposure were 1.84 (1.64, 2.06), 1.24 (1.19, 1.28) and 1.05 (1.04, 1.07), respectively; the correlation coefficient (*β*) and 95% *CI* of FPG in the highest quartile, per-quartile increase and continuous variable of ALAN exposure were 0.36 (0.32, 0.41), 0.14 (0.13, 0.16) and 0.04 (0.03, 0.05), respectively; and the corresponding *β* and 95% *CI* of insulin were −3.00 (−3.15, −2.84), −1.19 (−1.24, −1.14) and −0.37 (−0.40, −0.34), respectively. The *β* (95% *CI*) of HOMA-IR and HOMA-β were −0.22 (−0.25, −0.20) and −27.30 (−29.11, −25.48) for a per-quartile increase in ALAN, respectively. Further adjusting for WC, BMI, WHtR, WHR, VFI, or BFP, the associations of ALAN exposure with T2DM, FPG, insulin, HOMA-IR, and HOMA-β were steady in their direction and statistical significance. Given the role of environmental factors in T2DM, environmental covariates were incorporated into the adjusted models 10–17 in Table S1. The results showed that ALAN was independently associated with glucose metabolism and T2DM further adjusting for the air pollution and green space based on model 3, and the effect sizes remained robust.

**Table 2:** The associations of outdoor ALAN exposure with T2DM and glucose metabolism indexes

ALAN	Model 1	Model 2	Model 3	Model 4	Model 5	Model 6	Model 7	Model 8	Model 9
**T2DM (*OR*, 95%*CI*)**									

Q1 (≤0.21 nW/cm^2^/sr)	1.00	1.00	1.00	1.00	1.00	1.00	1.00	1.00	1.00
Q2 (0.21–0.57 nW/cm^2^/sr)	1.43 (1.28, 1.60)	1.50 (1.34, 1.67)	1.47 (1.31, 1.64)	1.46 (1.30, 1.63)	1.37 (1.22, 1.54)	1.46 (1.30, 1.63)	1.41 (1.26, 1.58)	1.37 (1.22, 1.53)	1.37 (1.23, 1.54)
Q3 (0.57–1.83 nW/cm^2^/sr)	2.17 (1.96, 2.41)	2.30 (2.07, 2.56)	2.20 (1.97, 2.45)	1.80 (1.62, 2.02)	1.92 (1.72, 2.15)	1.84 (1.65, 2.06)	1.94 (1.74, 2.17)	1.92 (1.72, 2.14)	1.91 (1.71, 2.13)
Q4 (>1.83 nW/cm^2^/sr)	1.84 (1.65, 2.05)	2.02 (1.81, 2.26)	1.84 (1.64, 2.06)	1.53 (1.36, 1.73)	1.60 (1.42, 1.79)	1.53 (1.36, 1.72)	1.60 (1.42, 1.80)	1.57 (1.39, 1.77)	1.58 (1.40, 1.77)
*P for trend*	<0.001	<0.001	<0.001	<0.001	<0.001	<0.001	<0.001	<0.001	<0.001
Per-quartile increase	1.24 (1.20, 1.28)	1.28 (1.24, 1.32)	1.24 (1.19, 1.28)	1.15 (1.11, 1.19)	1.18 (1.14, 1.22)	1.15 (1.11, 1.19)	1.18 (1.14, 1.22)	1.18 (1.14, 1.22)	1.17 (1.13, 1.22)
Continuous	1.05 (1.04, 1.07)	1.07 (1.06, 1.09)	1.05 (1.04, 1.07)	1.03 (1.01, 1.05)	1.04 (1.02, 1.06)	1.03 (1.01, 1.05)	1.04 (1.02, 1.06)	1.04 (1.02, 1.06)	1.04 (1.02, 1.06)

**FPG (*β*, 95%*CI*)**									

Q1 (≤0.21 nW/cm^2^/sr)	1.00	1.00	1.00	1.00	1.00	1.00	1.00	1.00	1.00
Q2 (0.21–0.57 nW/cm^2^/sr)	0.19 (0.15, 0.23)	0.21 (0.17, 0.25)	0.20 (0.16, 0.24)	0.20 (0.16, 0.24)	0.17 (0.13, 0.21)	0.21 (0.17, 0.25)	0.19 (0.15, 0.23)	0.18 (0.13, 0.22)	0.17 (0.13, 0.21)
Q3 (0.57–1.83 nW/cm^2^/sr)	0.51 (0.47, 0.55)	0.54 (0.50, 0.58)	0.52 (0.48, 0.56)	0.42 (0.38, 0.47)	0.45 (0.41, 0.49)	0.53 (0.48, 0.57)	0.47 (0.42, 0.51)	0.46 (0.41, 0.50)	0.45 (0.41, 0.49)
Q4 (>1.83 nW/cm^2^/sr)	0.36 (0.32, 0.40)	0.41 (0.36, 0.45)	0.36 (0.32, 0.41)	0.28 (0.23, 0.32)	0.29 (0.25, 0.34)	0.38 (0.34, 0.43)	0.32 (0.27, 0.36)	0.30 (0.26, 0.35)	0.30 (0.26, 0.35)
*P for trend*	<0.001	<0.001	<0.001	<0.001	<0.001	<0.001	<0.001	<0.001	<0.001
Per-quartile increase	0.15 (0.13, 0.16)	0.16 (0.15–0.17)	0.14 (0.13, 0.16)	0.11 (0.10, 0.12)	0.12 (0.11, 0.13)	0.15 (0.14, 0.16)	0.13 (0.11, 0.14)	0.12 (0.11, 0.14)	0.12 (0.11, 0.14)
Continuous	0.04 (0.03, 0.05)	0.05 (0.04, 0.06)	0.04 (0.03, 0.05)	0.03 (0.02, 0.03)	0.03 (0.02, 0.04)	0.04 (0.03, 0.05)	0.03 (0.03, 0.04)	0.03 (0.02, 0.04)	0.04 (0.04–0.05)

**Insulin (*β*, 95%*CI*)**									

Q1 (≤0.21 nW/cm^2^/sr)	1.00	1.00	1.00	1.00	1.00	1.00	1.00	1.00	1.00
Q2 (0.21–0.57 nW/cm^2^/sr)	−0.38 (−0.52, −0.24)	−0.40 (−0.54, −0.26)	−0.39 (−0.53, −0.25)	−0.40 (−0.54, −0.26)	−0.67 (−0.81, −0.53)	−0.42 (−0.56, −0.29)	−0.39 (−0.53, −0.25)	−0.68 (−0.82, −0.54)	−0.40 (−0.55, −0.26)
Q3 (0.57–1.83 nW/cm^2^/sr)	−3.23 (−3.37, −3.08)	−3.27 (−3.41, −3.12)	−3.20 (−3.35, −3.05)	−3.77 (−3.91, −3.62)	−3.75 (−3.90, −3.61)	−3.72 (−3.87, −3.58)	−3.21 (−3.36, −3.06)	−3.74 (−3.89, −3.60)	−3.21 (−3.36, −3.06)
Q4 (>1.83 nW/cm^2^/sr)	−2.98 (−3.13, −2.83)	−3.03 (−3.18, −2.88)	−3.00 (−3.15, −2.84)	−3.50 (−3.65, −3.35)	−3.57 (−3.72, −2.42)	−3.51 (−3.66, −3.36)	−3.00 (−3.15, −2.84)	−3.59 (−3.74, −3.44)	−3.01 (−3.17, −2.86)
*P for trend*	<0.001	<0.001	<0.001	<0.001	<0.001	<0.001	<0.001	<0.001	<0.001
Per-quartile increase	−1.21 (−1.25, −1.16)	−1.22 (−1.27, −1.17)	−1.19 (−1.24, −1.14)	−1.39 (−1.44, −1.34)	−1.39 (−1.44, −1.34)	−1.39 (−1.44, −1.34)	−1.19 (−1.24, −1.14)	−1.40 (−1.44, −1.35)	−1.20 (−1.25, −1.15)
Continuous	−0.41 (−0.44, −0.38)	−0.41 (−0.44, −0.38)	−0.37 (−0.40, −0.34)	−0.44 (−0.47, −0.41)	−0.43 (−0.45, −0.40)	−0.44 (−0.47, −0.41)	−0.38 (−0.41, −0.35)	−0.44 (−0.46, −0.41)	−0.38 (−0.41, −0.35)

**HOMA-IR (*β*, 95%*CI*)**									

Q1 (≤0.21 nW/cm^2^/sr)	1.00	1.00	1.00	1.00	1.00	1.00	1.00	1.00	1.00
Q2 (0.21–0.57 nW/cm^2^/sr)	0.03 (−0.02, 0.08)	0.04 (−0.01, 0.09)	0.30 (−0.02, 0.08)	0.03 (−0.02, 0.08)	−0.06 (−0.10, −0.01)	0.02 (−0.02, 0.07)	0.00 (−0.05, 0.05)	−0.06 (−0.10, −0.01)	−0.06 (−0.10, −0.01)
Q3 (0.57–1.83 nW/cm^2^/sr)	0.53 (−0.57, −0.48)	−0.52 (−0.57, −0.47)	−0.52 (−0.57, −0.47)	−0.72 (−0.76, −0.67)	−0.70 (−0.75, −0.65)	−0.70 (−0.75, −0.65)	−0.62 (−0.67, −0.57)	−0.70 (0.75, −0.65)	−0.70 (−0.75, −0.65)
Q4 (>1.83 nW/cm^2^/sr)	−0.53 (−0.58, −0.47)	−0.52 (−0.57, −0.47)	−0.54 (−0.59, −0.49)	−0.71 (−0.76, −0.66)	−0.72 (−0.77, −0.67)	−0.71 (−0.76, −0.66)	−0.64 (−0.69, −0.59)	−0.73 (−0.78, −0.68)	−0.72 (−0.77, −0.66)
*P for trend*	<0.001	<0.001	<0.001	<0.001	<0.001	<0.001	<0.001	<0.001	<0.001
Per-quartile increase	−0.22 (−0.23, −0.20)	−0.22 (−0.23, −0.20)	−0.22 (−0.25, −0.20)	−0.29 (−0.30, −0.27)	−0.28 (−0.30, −0.27)	−0.28 (−0.30, −0.27)	−0.26 (−0.27, −0.24)	−0.28 (−0.30, −0.27)	−0.28 (−0.30, −0.26)
Continuous	−0.08 (−0.09, −0.07)	−0.08 (−0.09, −0.07)	−0.07 (−0.08, −0.06)	−0.10 (−0.11, −0.09)	−0.09 (−0.10, −0.08)	−0.10 (−0.11, −0.09)	−0.09 (−0.10, −0.08)	−0.09 (−0.10, −0.08)	−0.09 (−0.10, −0.08)

**HOMA-β (*β*, 95%*CI*)**									

Q1 (≤0.21 nW/cm^2^/sr)	1.00	1.00	1.00	1.00	1.00	1.00	1.00	1.00	1.00
Q2 (0.21–0.57 nW/cm^2^/sr)	−18.41 (−23.62, −13.20)	−19.83 (−25.02, −14.63)	−19.90 (−25.12, −14.69)	−19.94 (−25.16, −14.72)	−20.76 (−26.00, −15.53)	−19.90 (−25.21, −14.77)	−20.02 (−25.24, −14.79)	−20.73 (−25.99, −14.47)	−20.87 (−26.13, 15.61)
Q3 (0.57–1.83 nW/cm^2^/sr)	−78.09 (−83.32, −72.86)	−80.12 (−85.35, −74.90)	−79.06 (−84.42, −73.69)	−80.26 (−85.69, −74.83)	−80.76(-86.18, −75.34)	−80.27 (−85.69, −74.85)	−79.35 (−84.74, −73.96)	−80.48 (−85.93, −75.04)	−80.77 (−86.23, −75.32)
Q4 (>1.83 nW/cm^2^/sr)	−67.93 (−73.39, −62.47)	−71.13 (−76.59, 65.67)	−69.82 (−75.51, −64.12)	−70.93 (−76.67, −65.18)	−71.52 (−77.28, −65.77)	−71.04 (−76.78, −65.29)	−70.17 (−75.89, −64.45)	−71.30 (−77.08, −65.52)	−71.44 (−77.23, −65.66)
*P for trend*	<0.001	<0.001	<0.001	<0.001	<0.001	<0.001	<0.001	<0.001	<0.001
Per-quartile increase	−27.01 (−28.73, −25.29)	−28.03 (−29.75, −26.31)	−27.30 (−29.11, −25.48)	−27.56 (−29.40, −25.72)	−27.83 (−29.66, −25.99)	−27.63 (−29.47, −25.79)	−27.39 (−29.21, −25.56)	−27.75 (−29.60, −25.91)	−27.78 (−29.63, −25.94)
Continuous	−8.91 (−9.93, −7.89)	−9.43 (−10.45, −8.41)	−8.44 (−9.49, −7.40)	−8.43 (−9.48, −7.38)	−8.48 (−9.53, −7.43)	−8.46 (−9.51, −7.41)	−8.53 (−9.58, −7.48)	−8.44 (−9.49, −7.38)	−8.43 (−9.49, −7.38)

ALAN: artificial light at night; *OR*, odds ratio; *β*, correlation coefficient; *CI*, confidence interval; T2DM: type 2 diabetes mellitus; FPG, fasting plasma glucose; HOMA-IR: insulin resistance index; HOMA-β: β-cell function index; WC: waist circumference; BMI, body mass index; WHtR: waist-to-height ratio; WHR: waist-to-hip ratio; VFI: visceral fat index; BFP: body fat percentage.

Model 1 was the crude model. Model 2 was further adjusted for age and gender. Model 3 was additionally adjusted for education status, marital status, per capita monthly income, smoking and drinking status, more vegetable and fruit intake, high-fat diet, physical activity, and family history of diabetes based on Model 2. Model 4 was additionally adjusted for WC based on Model 3. Model 5 was additionally adjusted for BMI based on Model 3. Model 6 was additionally adjusted for WHtR based on Model 3. Model 7 was additionally adjusted for WHR based on Model 3. Model 8 was additionally adjusted for VFI based on Model 3. Model 9 was additionally adjusted for BFP based on Model 3.

**Figure 1:**
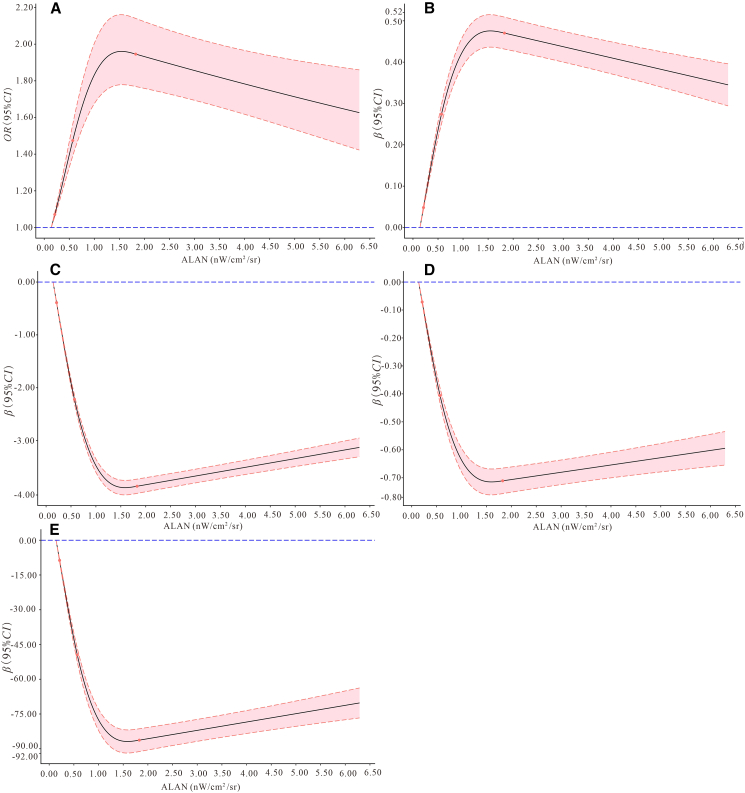
The relationship of outdoor ALAN exposure with T2DM and glucose metabolism indexes in the restricted cubic spline (A) T2DM; B: FPG; C: Insulin; D: HOMA-IR; E: HOMA-β. Abbreviations: OR, odds ratio; β, correlation coefficient; CI, confidence interval; ALAN, artificial light at night; T2DM: type 2 diabetes mellitus; FPG, fasting plasma glucose; HOMA-IR: insulin resistance index; HOMA-β: β-cell function index. The model was adjusted for age, gender, education status, marital status, per capita monthly income, smoking and drinking status, more vegetable and fruit intake, high-fat diet, physical activity, and family history of diabetes.

Figure 1 showed that the relationship between ALAN and T2DM/FPG was positive nonlinear, while the relationship between ALAN and insulin/HOMA-IR/HOMA-β was negative and nonlinear (All *P* for the overall association test were <0.001, and all *P* for the non-linear association test were <0.001. All *P* were displayed in Table S2).

### Sensitivity analyses

In the sensitivity analyses, the lag 0-year, lag 1-year, and lag 2-year averages of ALAN exposure were employed as the exposure variable, and the results implied comparable effect estimates for T2DM, FPG, and insulin (Table 3). For example, the *OR* (95%*CI*) for T2DM in the highest quartile, per-quartile increase, and continuous variable of ALAN in lag 0-year average were 1.88 (1.67, 2.12), 1.24 (1.19, 1.28), and 1.05 (1.04, 1.07), respectively. The corresponding *β*(95%*CI*) for FPG and insulin were 0.40 (0.35, 0.44), 0.15 (0.14,0.16), 0.04 (0.03, 0.05), and −2.84 (−3.03, −2.69), −1.19 (−1.25, −1.14), −0.37 (−0.40, −0.35), respectively. The corresponding *β* (95% *CI*) of HOMA-IR and HOMA-β were −0.22 (−0.24, −0.20) and −27.83 (−29.67, −25.99) for a per-quartile increase in lag 0-year average of ALAN, respectively.

**Table 3:** The associations of the average ALAN levels of different years with T2DM and glucose metabolism indexes

Variable	T2DM *OR* (95% *CI*)	FPG *β* (95% *CI*)	Insulin *β* (95% *CI*)	HOMA-IR *β* (95% *CI*)	HOMA-β *β* (95% *CI*)
**Lag 0-year**					

Q1 (≤0.25 nW/cm^2^/sr)	1.00	1.00	1.00	1.00	1.00
Q2 (0.25–0.56 nW/cm^2^/sr)	1.43 (1.27, 1.60)	0.19 (0.15, 0.24)	0.46 (0.31, 0.60)	0.23 (0.18, 0.38)	−11.20 (−16.50, −5.91)
Q3 (0.56–1.83 nW/cm^2^/sr)	2.06 (1.84, 2.31)	0.49 (0.45, 0.54)	−2.74 (−2.89, −2.59)	−0.42 (−0.47, −0.37)	−73.74 (−79.25, −68.23)
Q4 (>1.83 nW/cm^2^/sr)	1.88 (1.67, 2.12)	0.40 (0.35, 0.44)	−2.84 (−3.03, −2.69)	−0.49 (−0.55, −0.44)	−70.77 (−76.55, −64.98)
*P for trend*	<0.001	<0.001	<0.001	<0.001	<0.001
Per-quartile increase	1.24 (1.19, 1.28)	0.15 (0.14, 0.16)	−1.19 (−1.25, −1.14)	−0.22 (−0.24, −0.20)	−27.83 (−29.67, −25.99)
Continuous	1.05 (1.04, 1.07)	0.04 (0.03, 0.05)	−0.37 (−0.40, −0.35)	−0.07 (−0.08, −0.06)	−8.40 (−9.36, −7.44)

**Lag 1-year**					

Q1 (≤0.17 nW/cm^2^/sr)	1.00	1.00	1.00	1.00	1.00
Q2 (0.17–0.53 nW/cm^2^/sr)	1.06 (0.95, 1.18)	−0.02 (−0.06, 0.02)	−0.89 (−1.03, −0.74)	−0.24 (−0.28, −0.19)	−0.25 (−5.44, 4.94)
Q3 (0.53–1.82 nW/cm^2^/sr)	1.84 (1.65, 2.05)	0.40 (0.36, 0.45)	−3.64 (−3.79, −3.49)	−0.71 (−0.76, −0.66)	−69.75 (−75.29, −64.20)
Q4 (>1.82 nW/cm^2^/sr)	1.57 (1.41, 1.75)	0.27 (0.22, 0.31)	−3.25 (−3.40, −3.10)	−0.67 (−0.72, −0.62)	−60.29 (−65.80, −54.77)
*P for trend*	<0.001	<0.001	<0.001	<0.001	<0.001
Per-quartile increase	1.20 (1.16, 1.24)	0.12 (0.10, 0.13)	−1.23 (−1.28, −1.18)	−0.24 (−0.26, −0.23)	−24.57 (−26.34, −22.79)
Continuous	1.05 (1.04, 1.07)	0.04 (0.03, 0.05)	−0.37 (−0.40, −0.34)	−0.07 (−0.08, −0.06)	−8.35 (−9.44, −7.25)

**Lag 2-year**					

Q1 (≤0.22 nW/cm^2^/sr)	1.00	1.00	1.00	1.00	1.00
Q2 (0.22–0.62 nW/cm^2^/sr)	1.48 (1.32, 1.66)	0.17 (0.13, 0.21)	−0.58 (−0.73, −0.44)	−0.03 (−0.08, 0.03)	−24.19 (−29.53, −18.84)
Q3 (0.62–1.81 nW/cm^2^/sr)	2.41 (2.15, 2.70)	0.54 (0.50, 0.58)	−3.87 (−4.02, −3.71)	−0.69 (−0.74, −0.63)	−90.07 (−95.63, −84.51)
Q4 (>1.81 nW/cm^2^/sr)	1.92 (1.71, 2.16)	0.36 (0.32, 0.41)	−2.87 (−3.03, −2.72)	−0.50 (−0.55, −0.45)	−70.84 (−76.41, −65.28)
*P for trend*	<0.001	<0.001	<0.001	<0.001	<0.001
Per-quartile increase	1.24 (1.20, 1,28)	0.14 (0.13, 0.16)	−1.16 (−1.21, −1.11)	−0.21 (−0.23, −0.19)	−27.12 (−28.91, −25.33)
Continuous	1.05 (1.03, 1.07)	0.03 (0.03, 0.04)	−0.34 (−0.37, −0.31)	−0.07 (−0.08, −0.06)	−7.78 (−8.83, −6.73)

ALAN: artificial light at night; *OR*, odds ratio; *β*, correlation coefficient; *CI*, confidence interval; T2DM: type 2 diabetes mellitus; FPG, fasting plasma glucose; HOMA-IR: insulin resistance index; HOMA-β: β-cell function index; The model was adjusted for age, gender, education status, marital status, per capita monthly income, smoking and drinking status, more vegetable and fruit intake, high-fat diet, physical activity, and family history of diabetes.

### Stratified analysis

**Figure 2:**
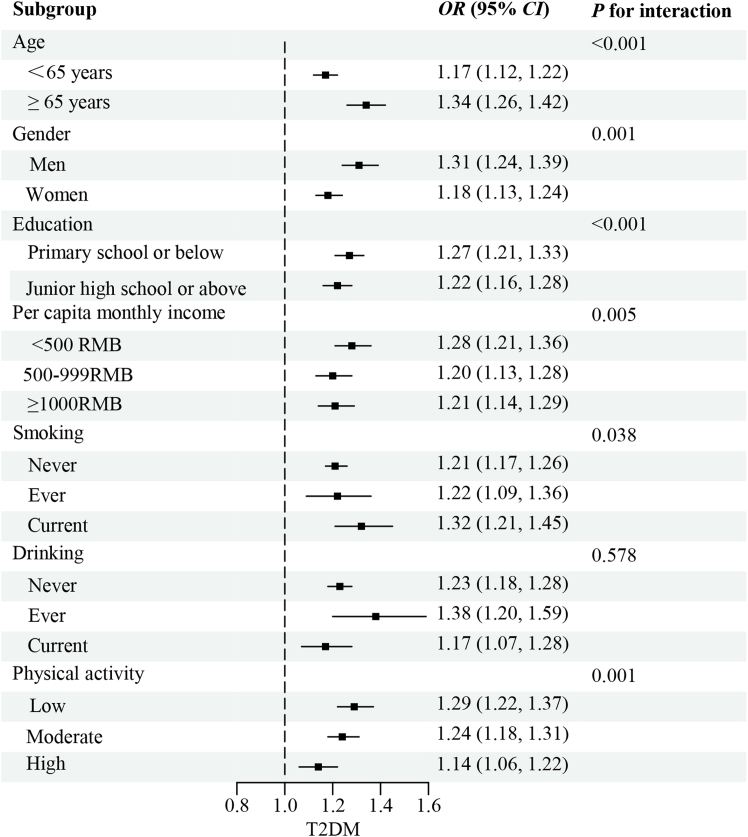
The stratified analysis of the association between outdoor ALAN exposure (per-quartile increment) with T2DM Abbreviations: *OR*, odds ratio; *CI*, confidence interval; ALAN, artificial light at night; T2DM, type 2 diabetes mellitus. The model was adjusted for age, gender, education status, marital status, per capita monthly income, smoking and drinking status, more vegetable and fruit intake, high-fat diet, physical activity, and family history of diabetes, except for the stratified variable.

The stratified analysis between the association of outdoor ALAN exposure (per-quartile increment) with T2DM is described in Figure 2. The effects of ALAN exposure with T2DM were stronger in participants ≥65 years old, men, current smokers, with a lower level of education, income, or physical activity. For instance, the larger effects of ALAN exposure on T2DM were found among participants ≥65 years old (*OR*: 1.34, 95%*CI*: 1.26, 1.42) compared with those <65 years old (*OR*: 1.17, 95%*CI*: 1.12, 1.22). Similar results of stratified analyses were found between ALAN exposure with glucose metabolism (Figures S1, S2, S3 and S4).

### Mediating role of obesity indices

**Figure 3:**
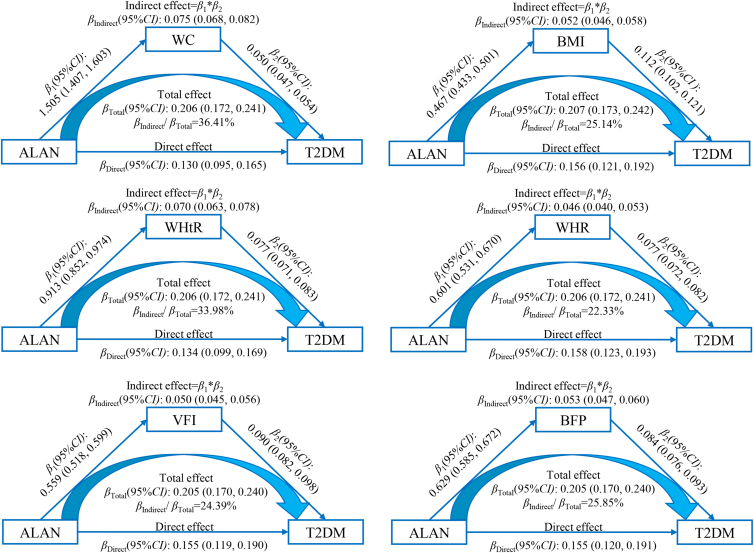
The mediation effect of obese anthropometric indices on the relationship between outdoor ALAN exposure (per-quartile increment) and T2DM Abbreviations: *β*, correlation coefficient; *CI*, confidence interval; ALAN, artificial light at night; T2DM, type 2 diabetes mellitus; WC, waist circumference; BMI, body mass index; WHtR, waist-to-height ratio; WHR, waist-to-hip ratio; VFI, visceral fat index; BFP, body fat percentage. Adjusted for age, gender, education status, marital status, per capita monthly income, smoking and drinking status, more vegetable and fruit intake, high-fat diet, physical activity, and family history of diabetes.

The associations of ALAN exposure with obesity indices, as well as the associations of those six obesity indices with FPG, insulin, HOMA-IR, HOMA-β, and T2DM in the mediation analyses, are shown in Tables S3. Figure 3 displays the association of outdoor ALAN exposure (per-quartile increment) with T2DM, which can be partly explained by increased WC, BMI, WHtR, WHR, VFI, and BFP, and the percentages for T2DM were estimated at 36.41%, 25.14%, 33.98%, 22.33%, 24.39%, and 25.85%, respectively. Detailed data were presented in Table S3. In addition, the mediation effects of obesity indices on the associations of outdoor ALAN exposure (per-quartile increment) with FPG, insulin, and HOMA-IR were estimated at 8.43%–31.19%, and the detailed data are shown in Figures S5, S6, and S7 and Table S3. For HOMA-β, only BMI mediated the association, and the percentage was 2.07% (Figure S8).

## Discussion

This study comprehensively explored the associations of chronic exposure to outdoor ALAN with T2DM and the mediating effects of obesity in a larger rural population. The findings highlighted that outdoor ALAN exposure was linked to the increased prevalence of T2DM, elevated FPG level, and lower fasting insulin, HOMA-IR, and HOMA-β. Furthermore, the elderly, men, current smokers, individuals with lower socioeconomic status or physical activity were more vulnerable to the adverse health effects of ALAN. Mediation analyses shed the associations of ALAN exposure with T2DM and glucose metabolism were partially mediated by WC, BMI, WHtR, WHR, VFI, and BFP.

Consistent with previous studies, the current results verified the detrimental role of outdoor ALAN exposure in metabolic health. Our study found that exposure to higher outdoor ALAN levels was linked to the increased prevalence of T2DM and impaired glucose metabolism in rural areas after adjusting for potential confounding factors. Further adjusting for obesity indices (WC, BMI, WHtR, WHR, VFI, or BFP), air pollution, and green space, the associations of ALAN exposure with T2DM, FPG, insulin, HOMA-IR, and HOMA-β were robust in their direction and statistical significance. The restricted cubic splines showed that the relationships between ALAN and T2DM/FPG were positive nonlinear, while the relationships between ALAN and insulin/HOMA-IR/HOMA-β were negative nonlinear. Furthermore, the sensitivity analyses implied the relationships remained steady with average ALAN exposure levels of different years as the independent variable. Although some studies have investigated the associations of chronic exposure to ALAN with T2DM and FPG in China, the studies explored the associations in rural areas with limited resource is rare.^15^^,^^16^^,^^29^^,^^30^ Only one study conducted by Wen et al. referred to the rural areas using data from the China Family Panel Studies (CFPS) and the China Health and Retirement Longitudinal Study (CHARLS) with 28,903 participants followed for 3 years showed that there were regional differences in the association between ALAN exposure and diabetes risk as positive association in rural areas and negative association in urban areas.^7^ The phenomenon suggests that further studies are warranted to verify the association. Meanwhile, the results of this study supported the positive association between outdoor ALAN exposure and T2DM in rural areas, which implied that the adverse health effects of outdoor ALAN exposure should be given more attention, and the targeted public health interventions to reduce outdoor ALAN exposure are needed in rural areas, such as optimizing the design of street lamp, eliminating unnecessary lighting facilities, use induction lights in public areas, and avoiding excessive use of light in daily life. Furthermore, this is the first study to explore the association between outdoor ALAN exposure and fasting insulin in the T2DM population, and a negative relationship was found in this study. The potential mechanism underlying the negative correlation might be that exposure to ALAN disrupts the circadian rhythm by suppressing melatonin secretion, further influences the insulin secretory pathway (for example, incretin stimulation, synthesis, processing, and granule maturation), and leads to decreased insulin secretion.^11^^,^^12^^,^^14^^,^^31^^,^^32^^,^^33^ In addition, the correlation of ALAN with HOMA-IR and HOMA-β was both negative. The negative correlations of ALAN with insulin and HOMA-IR are intriguing findings. The inconsistent results of existing studies between ALAN and HOMA-IR remained. A prospective cohort study of 6730 pregnant women showed that there were positive associations of outdoor ALAN with insulin and HOMA-IR during the first trimester, but there was no significant association during the second trimester.^34^ The study of Zheng et al. showed that outdoor light at night exposure levels were positively associated with HOMA-IR and negatively associated with HOMA-β based on a national cross-sectional study in China.^16^ In the current cross-sectional study, negative associations of ALAN with insulin, HOMA-IR, and HOMA-β were found. Moreover, the restricted cubic splines showed that the relationships between ALAN and insulin/HOMA-IR/HOMA-β were negative nonlinear in the current study. Only the density of ALAN was considered in nearly all the published articles; information on different spectral bands (color) may contribute to the inconsistence.^14^ Further studies are warranted to confirm the findings of the current study and explore the underlying causes.

Prior studies have reported that the associations between outdoor ALAN exposure and T2DM or glucose metabolism indicators remained statistically significant despite adjusting for age, gender, education level, smoking, drinking, household income, physical activity, family history of diabetes, and BMI, which suggested that ALAN exposure might be an independent influencing factor of glucose metabolism.^16^^,^^35^ Moreover, the health effects of ALAN were modified by age.^36^ Thus, the stratified analyses were conducted and the results showed that the elderly, men, current smokers, participants with lower education, income, or physical activity might be the susceptible population to the adverse effects of ALAN exposure. The possible reason for the differences in age might be that the elderly were more likely to have sleep problems which is the leading risk factor of T2DM, and have a reduction in the ability of the clearance effect of ALAN, thus susceptible to the adverse health effects of ALAN exposure.^37^^,^^38^ The current study found an interesting phenomenon that men were more susceptible to the detrimental health effects of ALAN exposure than women. This observation was consistent with a previous epidemiological study in school-aged children and adolescents, which found that the association of ALAN exposure with BMI Z-scores was higher in boys compared with girls.^39^ Nevertheless, gender-specific associations between ALAN exposure and insulin were not found in the current study. Similarly, a previous study in adults indicated that ALAN exposure could increase the risk of T2DM in both men and women.^30^ Thus, it is necessary to explore the differences in the gender-specific associations among children, adolescents, and adults in further studies. Our study also found that people with a lower level of education or per capita monthly income, and smokers may have a greater risk of T2DM. This could be explained by the fact that those people might experience higher stress and have worse living conditions, both of which have been linked to T2DM.^40^^,^^41^^,^^42^ Regular exercise can consume body energy, stimulate the production of melatonin, which is conducive to the regulation of the body’s circadian rhythm, which might ultimately decrease the risk of T2DM.^43^ The present study helped to generate hypotheses that high-risk individuals with T2DM might benefit from controlling outdoor ALAN exposure. Meanwhile, these findings implied that the elderly, men, and individuals with lower socioeconomic status and unhealthy lifestyles should be prioritized as the management population to reduce the burden of T2DM in response to higher outdoor ALAN exposure levels.

It is equally crucial to explore the potential mechanisms of the epidemiological associations. Previous studies indicated some clues of the potential mechanisms of ALAN-causing diabetes.^13^^,^^44^^,^^45^ An increment of body weight might be a key approach. Previous experiments observed an increment of body weight in regular mice, TALLYHO mice, and hamsters after exposure to ALAN, as well as the plasma glucose levels.^13^^,^^44^^,^^45^ Epidemiological study on population also found ALAN was positively associated with obesity.^46^ Moreover, the previous studies including the prospective cohort study and a Mendelian randomization analysis, have shown that obesity, defined by different indicators, has causal effects on T2DM.^21^^,^^47^ Thus, obesity indicators were determined as mediating factors in this study. The results of mediation analyses displayed that obese anthropometric indices, including WC, BMI, WHtR, WHR, VFI, and BFP, played important mediation roles in the relationships of ALAN exposure with T2DM, FPG, insulin, and HOMA-IR through epidemiological approaches, regardless of the types of obese indices. It suggested potential pathways of ALAN-caused diabetes in the Chinese population, providing evidence that weight management may be an important measure in reducing the burden of T2DM related to ALAN exposure. As the framework of the cross-sectional study, the causality of the mediation analysis cannot be established in this study.

### Conclusion

This study revealed that chronic exposure to outdoor ALAN was positively associated with T2DM and impaired glucose metabolism in the rural adult population, particularly among the elderly, men, those with lower socioeconomic status, and unhealthy lifestyles. Obesity appears to partially mediate these associations. These findings emphasize the need for targeted public health interventions to reduce outdoor ALAN exposure in rural areas, and high-risk individuals, and weight management may be effective in reducing the burden of T2DM related to ALAN exposure. However, further studies are needed to validate these results.

### Limitations of the study

The current study comprehensively evaluated the associations of outdoor ALAN exposure with T2DM and the mediation effects of obesity in a large rural population. The strict survey design, adjusting for plenty of potential confounding factors as much as possible, as well as the sensitivity analyses of different years’ average exposures, ensured the reliability of the analysis. However, there were some limitations in this study. Firstly, the exposure level of outdoor ALAN was assessed based on satellite data, which differed from actual personal exposures. Secondly, even though many potential covariates were adjusted in this study, the associations of ALAN exposure with T2DM and impaired glucose metabolism might be influenced by other potential factors, such as indoor lightning appliances and night shift work. Thirdly, the cross-sectional design of the current research made it impossible to establish causal associations of ALAN exposure with T2DM and glucose metabolism, as well as the mediating effects of obesity in the association. Lastly, only the density of ALAN was considered in the current study; the effect of different spectral bands (color) on health should be further studied.

## Resource availability

### Lead contact

Further information and requests for resources should be directed to and will be fulfilled by the lead contact, Chongjian Wang (tjwcj2008@zzu.edu.cn).

### Materials availability

This study did not generate new unique reagents.

### Data and code availability

• The complete original data reported in this study cannot be deposited in a public repository. To request access, contact Professor Chongjian Wang (tjwcj2008@zzu.edu.cn).
• Code: This article does not report the original code.
• Any additional information required to reanalyze the data reported in this article is available from the lead contact upon request.

## Acknowledgments

This research was supported by the Noncommunicable Chronic Diseases-National Science and Technology Major Project (Grant NO: 2024ZD0531600), the 10.13039/501100006407Natural Science Foundation of Henan Province (Grant NO: 23230042151), and the Foundation of National Key Program of Research and Development of China (Grant NO: 2016YFC0900803). The funders had no role in the study design, data collection and analysis, decision to publish, or preparation of the article.

The authors thank all of the participants, coordinators, and administrators for their support and help during the research.

## Author contributions

C.W. designed the study. Xiaotian Liu, Y.Y., R.L., W.L., X.D., W.H., and J.H. conducted the study. Xiaotian Liu, Z.D., and K.Z. analyzed the data and took responsibility for the integrity and accuracy of the information. H.L., Xin Liu, and K.Z. contributed to the reagents/materials/analysis tools. Xiaotian Liu drafted and revised the manuscript. All authors reviewed the manuscript. All authors have approved the final manuscript.

## Declaration of interests

The authors declare no competing interests.

## STAR★Methods

### Key resources table

**Table:** 

REAGENT or RESOURCE	SOURCE	IDENTIFIER
**Biological samples**		

Blood from participants	Participants enrolled	NA

**Critical commercial assays**		

Fasting blood glucose	Roche glucose reagent	Cobas c501, Roche, Switzerland
Height	Participants enrolled	Manufactured instrument (tape)
Waist circumference	Participants enrolled	Manufactured instrument (tape)
Hip circumference	Participants enrolled	Manufactured instrument (tape)
Weight	Participants enrolled	V. BODY HBF-371, OMRON, Japan
Body fat percentage	Participants enrolled	V. BODY HBF-371, OMRON, Japan
Visceral fat index	Participants enrolled	V. BODY HBF-371, OMRON, Japan
Artificial light at night	The Visible and Infrared Imaging Suite cloud-free Day Night Band product	https://eogdata.mines.edu

**Software and algorithms**		

R	The R Foundation for Statistical Computing	V.4.4.0
SPSS	Statistical Product and Service Solutions	27.0

### Experimental model and study participant details

#### Study settings and participants

The participants of this study were derived from the Henan Rural Cohort (Registration number: ChiCTR-OOC-15006699). The detailed description of this cohort study was published previously.^48^^,^^49^ Briefly, a total of 39,259 participants aged 18-79 years were recruited at the baseline study from July 2015 to September 2017. After excluding participants with missing information on T2DM (n=63), FPG (n=8), fasting insulin (n=742), participants with cancer (n=321) and kidney failure (n=17), type 1 diabetes mellitus (n=3), a total of 38,105 participants were finally included in the analysis. The flowchart of participant selection was shown in Figure S9.

#### Ethics

The study was approved by the Life Science Ethics Committee of Zhengzhou University ([2015] MEC (S128)) and performed in accordance with the Declaration of Helsinki. The written informed consent was obtained from all participants before the survey.

### Method details

#### Outdoor ALAN exposure assessment

In this study, ALAN data were downloaded from the Visible and Infrared Imaging Suite cloud-free Day Night Band product (https://eogdata.mines.edu). The ALAN data had been available at ∼500m resolution on a monthly basis since 2012. The higher ALAN value, the stronger the brightness. The ALAN unit is radiance in nano watts per square centimeter per steradian (nW/cm^2^/sr). The value ranged from 0 to 472.632. Zero value meant no light, and larger values indicated stronger light radiance. Monthly ALAN values were extracted for each cohort participant based on the grid cell where their residential addresses were located. The annual exposure to ALAN was calculated for each participant by aggregating monthly values over one year prior to each participant’s visit date. The average ALAN exposure of participants in three-year prior to the baseline survey was considered as the chronic exposure to ALAN.

#### Obesity indices

The subjects’ shoes off and wearing light clothing were required for all measurement of obesity indices. The measurement of height, weight, WC, and hip circumference were conducted according to a standard protocol from the Department of Disease Control Ministry of Health of China.^25^ Height was measured in an upright position against a calibrated ruler. WC and hip circumference were measured at 1.0 centimeter above the navel and the maximal level of the hip, respectively. Height, WC, and hip circumference were measured to the nearest 0.1 cm. In accordance with the instrument operation instructions of, weight, VFI and BFP were measured by using a bioelectrical impendence analysis device (V. BODY HBF-371, OMRON, Japan). Weight was measured to the nearest 0.1 kg. Detail information on obese anthropometric measurements has been published elsewhere.^48^^,^^50^ BMI was estimated using the body weight (kg) divided by the square of height (m^2^). WHtR was generated as WC (cm) divided by height (cm), and WHR was evaluated as WC (cm) divided by hip circumference (cm).^23^^,^^24^^,^^25^^,^^26^^,^^27^^,^^28^^,^^50^^,^^51^

#### Covariates

All information was obtained from the participants by face-to-face interviews using a standardized questionnaire by well-trained investigators. The covariates included: age, gender, marital status (married/cohabiting, widowed/single/divorced/separation), educational level (primary school or below, junior high school or above), per capita monthly income (< 500 RMB, 500-999 RMB, and ≥ 1000 RMB), smoking and drinking (never, ever and current), more vegetable and fruit intake (no, yes), high-fat diet (no, yes), level of physical activity (low, moderate, and high),^49^^,^^50^^,^^51^ and family history of diabetes.

#### Other environmental variables

Environmental covariates including air pollution and green space were incorporated into this study. The data for particulate matter with aerodynamic diameters ≤1.0 μm (PM_1_), ≤2.5 μm (PM_2.5_), and ≤10 μm (PM_10_) across China during 2005-2016 were estimated using aerosol optical depth (AOD; from the MODIS satellite), meteorological data, land use information and other predictors. Daily concentration of nitrogen dioxide (NO_2_) was estimated with satellite-derived OMI data (Daily Level-3 Nitrogen Dioxide Product) and other predictors. Green space covariates including Enhanced Vegetation Index (EVI) and Normalized Difference Vegetation Index (NDVI) were sourced from the 16-day Moderate-resolution Imaging Spectroradiometer (MODIS) satellite images. The detailed method was described in elsewhere.^50^^,^^51^^,^^52^ The 3-year average exposure to PMs, NO_2_, as well as EVI/NDVI (within the buffer radiuses of 500 m) before the baseline survey were employed in the adjusted models.

#### Outcome assessment

After overnight fasting (more than eight hours), the blood samples of the participants were collected. FPG and fasting insulin were measured by the hexokinase method and radioimmunoassay using automatic biochemical analyzers, respectively (Cobas c501, Roche, Switzerland; GC-gamma radio immune assay counter, USTCZONKIA, China). T2DM patients were determined by the following definitions: self-reported diagnosed with T2DM previously by doctors and had been prescribed hypoglycemic drugs in the past two weeks^53^; or FPG ≥ 7.0 mmol/L. The insulin resistance index (HOMA-IR) was calculated as FPG × insulin/22.5, and β-cell function index (HOMA-β) was estimated as 20×insulin/(FPG-3.5).^54^

### Quantification and statistical analysis

Descriptive analyses were carried out to display the characteristics of the participants. Mean with standard deviation as well as frequencies with percentages were used to describe continuous variables and categorical variables, respectively. T-test and Chi-squared test were performed to test the intergroup differences. Outdoor ALAN exposure level was stratified into four levels according to quartiles. Multiple logistic regression as well as the generalized linear model were employed to assess the associations of ALAN exposure (as continuous variable and categorical variable) with T2DM, FPG, insulin, HOMA-IR, as well as HOMA-β, and effect sizes were described as *OR*, *β* and 95% *CI*. Previous studies have shown that age, sex, education status, marital status, per capita monthly income, smoking and drinking status, more vegetable and fruit intake, high-fat diet, physical activity, family history of diabetes, and obesity were associated with T2DM.^20^^,^^55^^,^^56^ Therefore, those covariates were selected in the adjustment. Nine models were developed in the main text as follows: Model 1 was the crude model; Model 2 was further adjusted for age and gender; Model 3 was additionally adjusted for education status, marital status, per capita monthly income, smoking and drinking status, more vegetable and fruit intake, high-fat diet, physical activity, and family history of diabetes based on Model 2; Model 4 was additionally adjusted for WC based on Model 3; Model 5 was additionally adjusted for BMI based on Model 3; Model 6 was additionally adjusted for WHtR based on Model 3; Model 7 was additionally adjusted for WHR based on Model 3; Model 8 was additionally adjusted for VFI based on Model 3; Model 9 was additionally adjusted for BFP based on Model 3. Given the role of environmental factors in T2DM, environmental covariates were incorporated into the adjusted models 10-17 in the Table S1. Model 10 was additionally adjusted for PM_1_ and EVI based on Model 3. Model 11 was additionally adjusted for PM_1_ and NDVI based on Model 3. Model 12 was additionally adjusted for PM_2.5_ and EVI based on Model 3. Model 13 was additionally adjusted for PM_2.5_ and NDVI based on Model 3. Model 14 was additionally adjusted for PM_10_ and EVI based on Model 3. Model 15 was additionally adjusted for PM_10_ and NDVI based on Model 3. Model 16 was additionally adjusted for NO_2_ and EVI based on Model 3. Model 17 was additionally adjusted for NO_2_ and NDVI based on Model 3. The restricted cubic spline was performed to explore the exposure-response relationships between ALAN exposure and T2DM as well as glucose metabolism indices based on three knots (25th, 50th, and 75th percentiles) of ALAN exposure.

To assess the stability of the effect estimates, a variety of lags of ALAN exposure levels including lag 0 (the present year), lag 1 (the previous year), and lag 2 (the previous two years) were employed in Model 3 as the exposure variables for the sensitivity analyses. To examine whether the associations varied by stratified variables, the stratified analyses were employed to assess the associations between ALAN exposure (per-quartile increment) with T2DM and glucose metabolism by age (<65, ≥65 years), gender (men, women), educational levels (primary school or below, junior high school or above), per capita monthly income (<500, 500-999, ≥1000RMB), smoking and drinking status (never, ever, current), and physical activity (low, moderate, high) in Model 3 except for stratified variable. The interactions were conducted to test the effect modification in subgroup analyses with a cross-product term included in the regression model (i.e., ALAN ∗age or ALAN ∗sex). As for the mediation analyses, WC, BMI, WHtR, WHR, VFI, and BFP were determined as mediators^44^^,^^45^^,^^46^^,^^47^ and the analyses were done using PROCESS of SPSS in Model 3. The mediation effect percentage of each mediator was generated as: (indirect effect/total effect) × 100%.

All data analyses were conducted by R software (version 4.2.3) and SPSS software (IBM-SPSS Inc., Armonk, NY, version 27.0). The statistical significance was considered with a two-tailed *P* < 0.05.

### Additional resources

This work is not part of or involving a clinical trial.
