# Supplementary material for: Obesity mediates the association of outdoor artificial light at night with type 2 diabetes mellitus
Source: iScience. 2026 Feb 26;29(3):115036. doi: 10.1016/j.isci.2026.115036 (PMC12999289; doi:10.1016/j.isci.2026.115036)
Supplement: Document S1. Figures S1–S9 and Tables S1–S3 [file mmc1.pdf]

iScience, Volume *29*

Supplemental information

Obesity mediates the association of outdoor artificial light at night with type 2 diabetes mellitus

Xiaotian Liu, Zhongao Ding, Yinghao Yuchi, Ruiying Li, Wei Liao, Xiaokang Dong, Wenqian Huo, Jian Hou, Hualiang Lin, Xin Liu, Kai Zhang, and Chongjian Wang

Subgroup	*β* (95% *CI*)	*P* for interaction
Age		<0.001
＜65 years	0.12 (0.10, 0.14)	
≥ 65 years	0.18 (0.15, 0.21)	
Gender		<0.001
Men	0.17 (0.15, 0.19)	
Women	0.11 (0.09, 0.13)	
Education		<0.001
Primary school or below	0.17 (0.15, 0.19)	
Junior high school or above	0.13 (0.11, 0.15)	
Per capita monthly income		<0.001
<500 RMB	0.17 (0.15, 0.20)	
500-999RMB	0.12 (0.10, 0.15)	
≥1000RMB	0.13 (0.11, 0.16)	
Smoking		<0.001
Never	0.13 (0.11, 0.15)	
Ever	0.14 (0.09, 0.19)	
Current	0.19 (0.16, 0.22)	
Drinking		0.373
Never	0.14 (0.12, 0.16)	
Ever	0.15 (0.08, 0.22)	
Current	0.15 (0.12, 0.19)	
Physical activity		<0.001
Low	0.18 (0.15, 0.20)	
Moderate	0.13 (0.11, 0.15)	
High	0.12 (0.09, 0.14)	

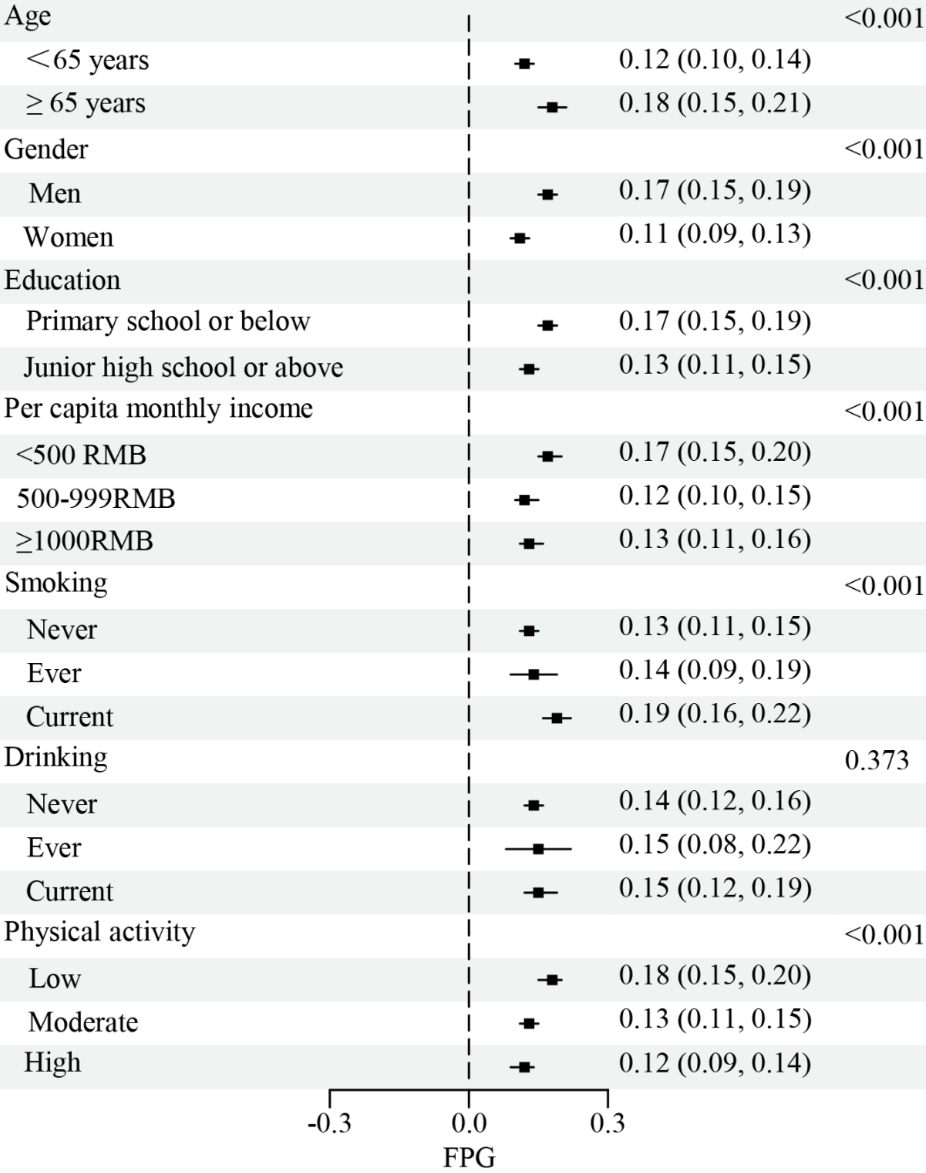

Supplemental Figure 1. The stratified analysis of the association between outdoor ALAN exposure (per-quartile increment) with FPG

Abbreviations: *β*, correlation coefficient; *CI*, confidence interval; ALAN, artificial light at night; FPG, fasting plasma glucose.

The model was adjusted for age, gender, education status, marital status, per capita monthly income, smoking and drinking status, more vegetable and fruit intake, high-fat diet, physical activity, and family history of diabetes except for stratified variable.

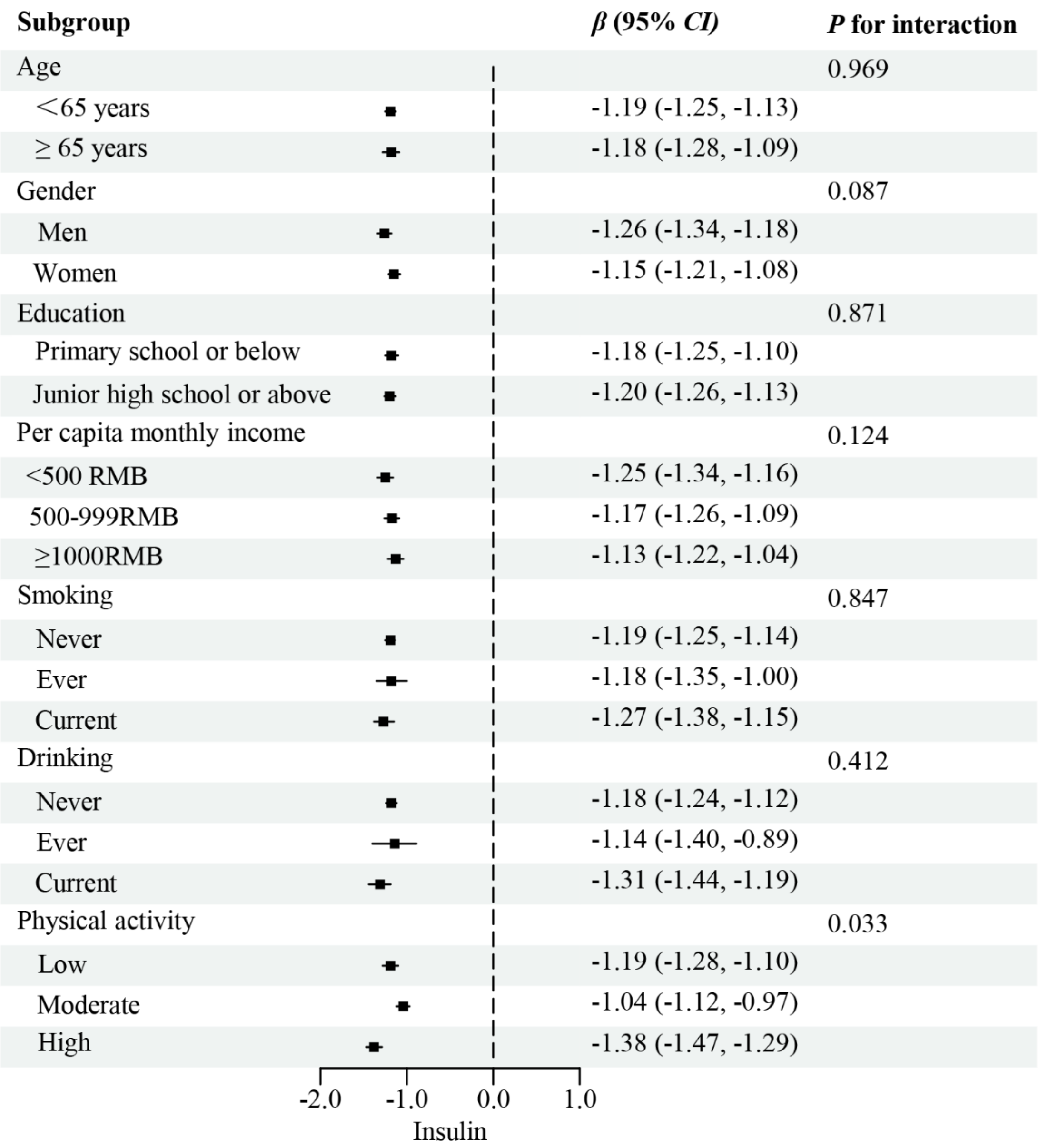

Supplemental Figure 2. The stratified analysis of the association between outdoor ALAN exposure (per-quartile increment) with insulin

Abbreviations: *β*, correlation coefficient; *CI*, confidence interval; ALAN, artificial light at night. The model was adjusted for age, gender, education status, marital status, per capita monthly income, smoking and drinking status, more vegetable and fruit intake, high-fat diet, physical activity, and family history of diabetes except for stratified variable.

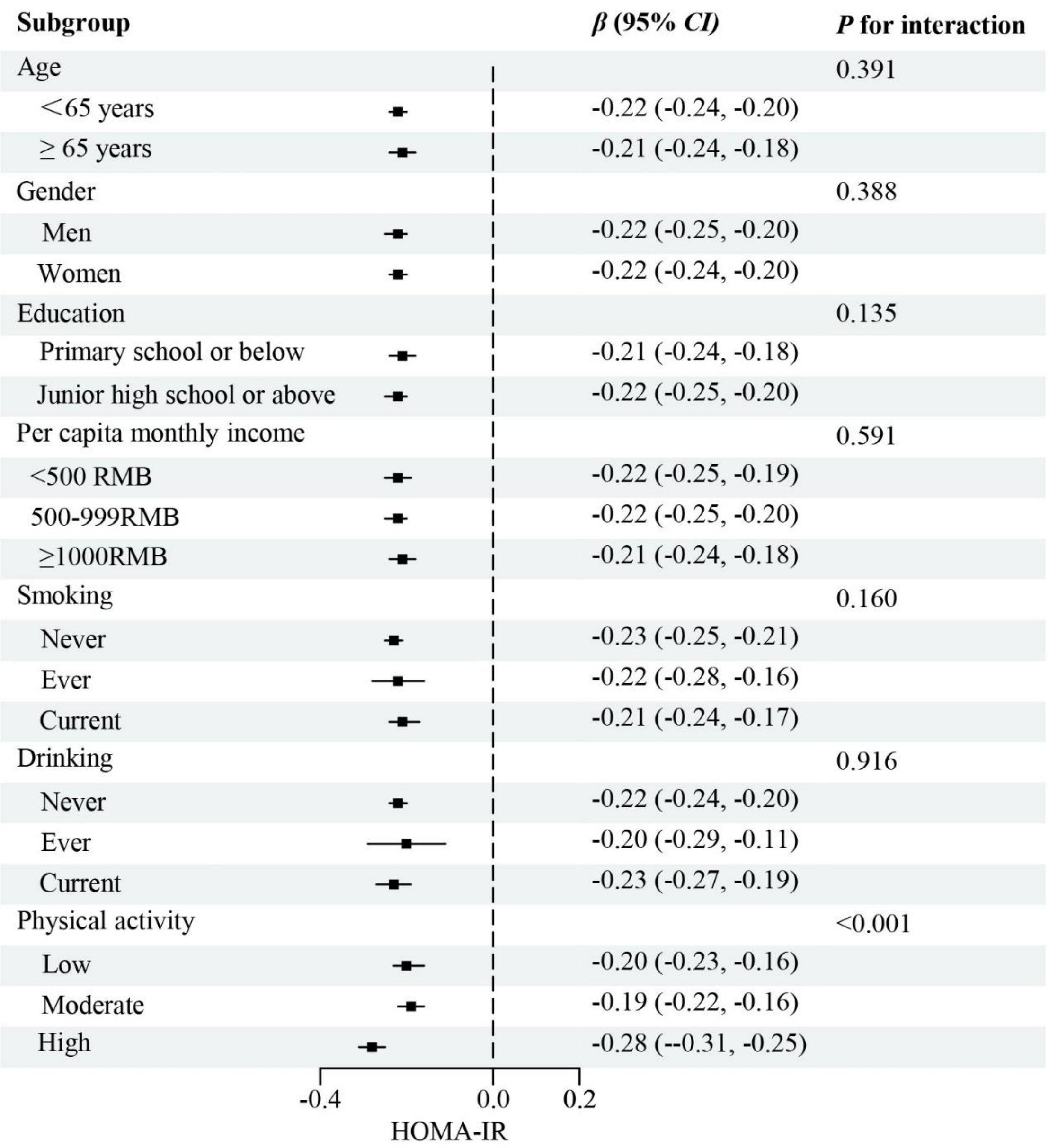

Supplemental Figure 3. The stratified analysis of the association between outdoor ALAN exposure (per-quartile increment) with HOMA-IR

Abbreviations: *β*, correlation coefficient; *CI*, confidence interval; ALAN, artificial light at night; HOMA-IR: insulin resistance index.

The model was adjusted for age, gender, education status, marital status, per capita monthly income, smoking and drinking status, more vegetable and fruit intake, high-fat diet, physical activity, and family history of diabetes except for stratified variable.

Subgroup	β (95% CI)	P for interaction
Age		0.547
＜65 years	-26.35 (-28.45, -24.25)	
≥ 65 years	-27.72 (-31.37, -24.07)	
Gender		<0.001
Men	-32.36 (-35.88, -28.84)	
Women	-22.85 (-24.79, -20.91)	
Education		0.425
Primary school or below	-27.57 (-30.57, -24.56)	
Junior high school or above	-26.13 (-28.34, -23.90)	
Per capita monthly income		0.119
<500 RMB	-28.43 (-32.09, -24.78)	
500-999RMB	-26.63 (-29.53, -23.74)	
≥1000RMB	-24.57 (-27.35, -21.80)	
Smoking		<0.001
Never	-24.42 (-26.16, -22.68)	
Ever	-25.62 (-29.55, -21.70)	
Current	-35.46 (-42.15, -28.77)	
Drinking		<0.001
Never	-25.17 (-27.04, -23.31)	
Ever	-27.09 (-32.21, -21.73)	
Current	-32.96 (-39.01, -26.91)	
Physical activity		0.292
Low	-27.47 (-30.75, -24.19)	
Moderate	-25.09 (-27.75, -22.43)	
High	-27.34 (-31.04, -23.63)	

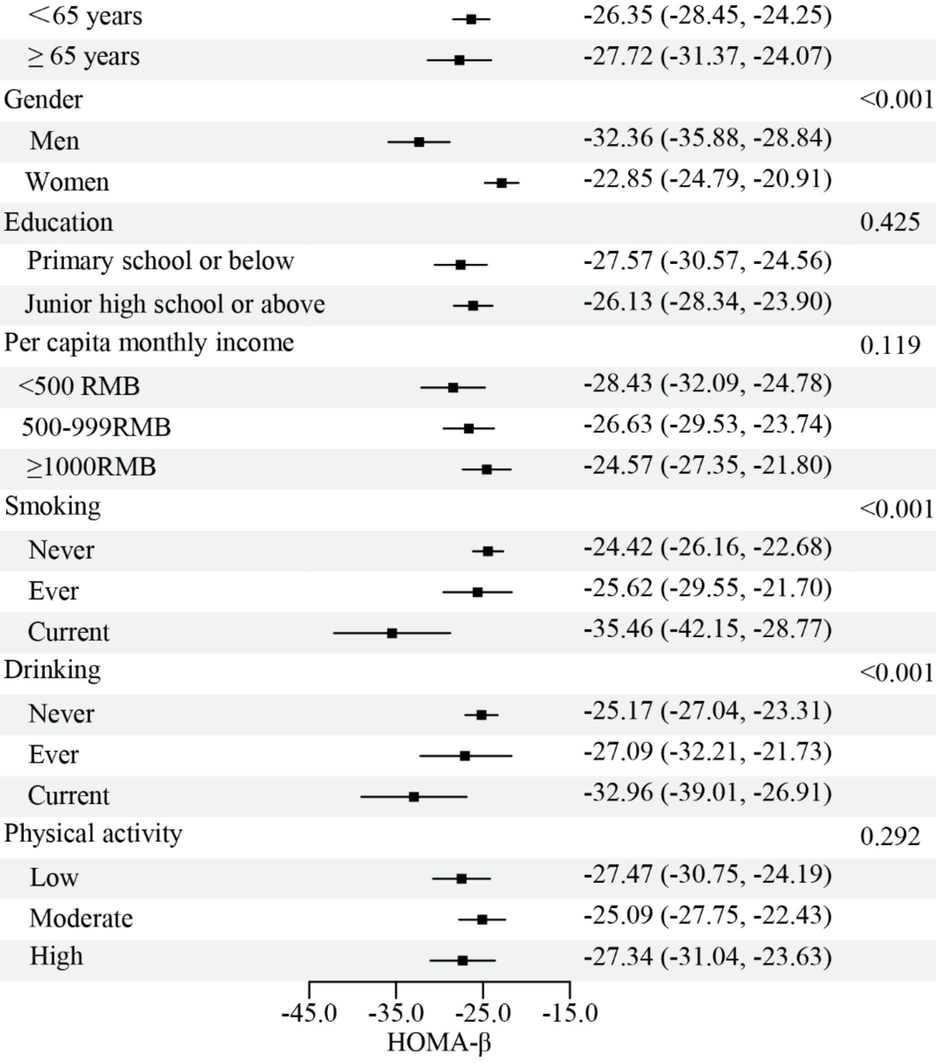

Supplemental Figure 4. The stratified analysis of the association between outdoor ALAN exposure (per-quartile increment) with HOMA-β

Abbreviations: *β*, correlation coefficient; *CI*, confidence interval; ALAN, artificial light at night; HOMA-β: β-cell function index.

The model was adjusted for age, gender, education status, marital status, per capita monthly income, smoking and drinking status, more vegetable and fruit intake, high-fat diet, physical activity, and family history of diabetes except for stratified variable.

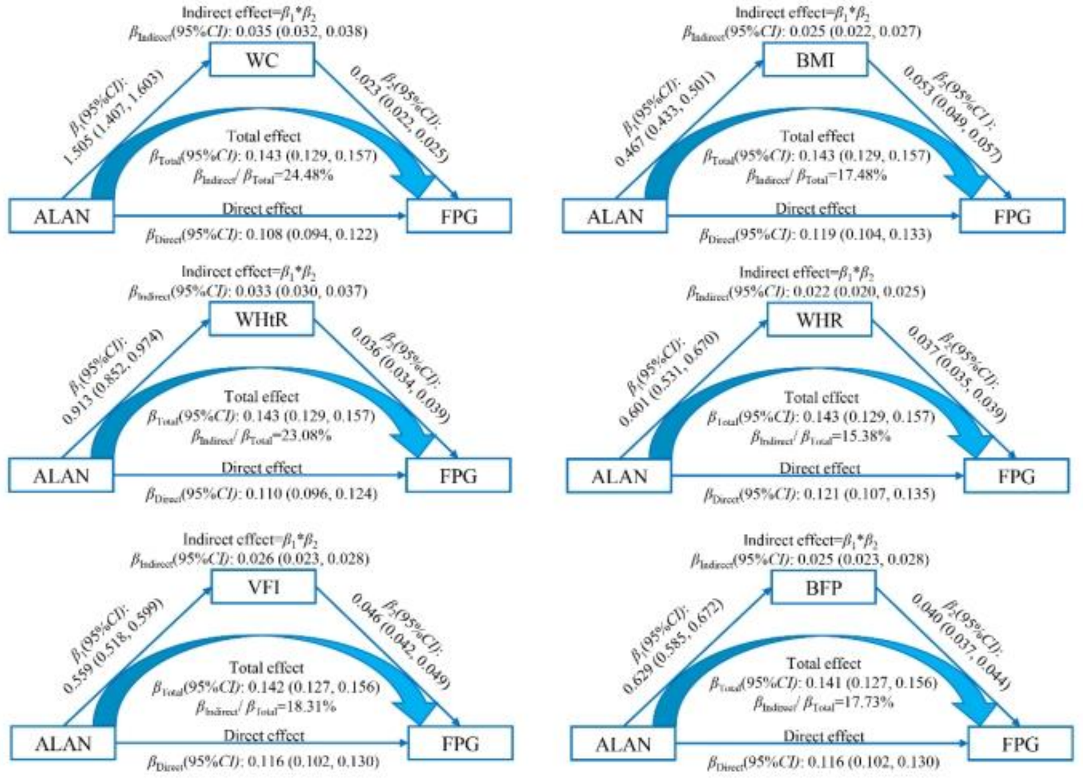

Supplemental Figure 5. The mediation effect of obese indices on the association between outdoor ALAN exposure (per-quartile increment) and FPG.

Abbreviations: β, correlation coefficient; *CI*, confidence interval; ALAN, artificial light at night; FPG, fasting plasma glucose; WC, waist circumference; BMI, body mass index; WHtR, waist-to-height ratio; WHR, waist-to-hip ratio; VFI, visceral fat index; BFP, body fat percentage.

Adjusted for age, gender, education status, marital status, per capita monthly income, smoking and drinking status, more vegetable and fruit intake, high-fat diet, physical activity, and family history of diabetes.

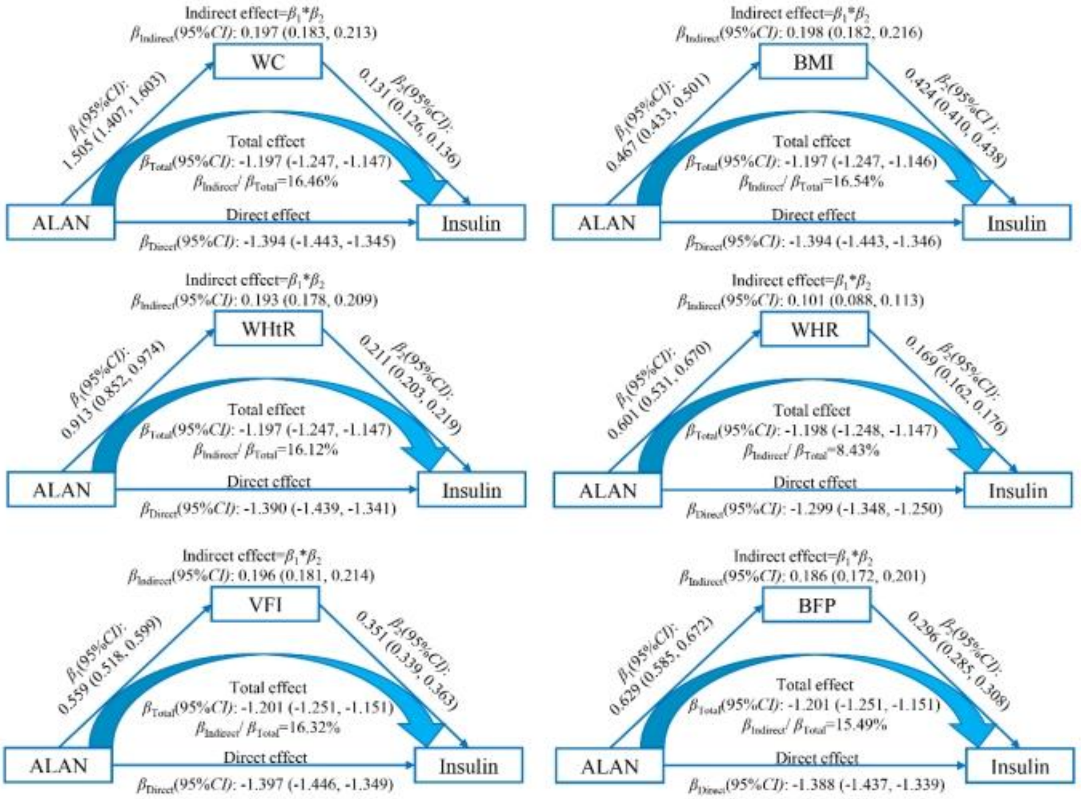

Supplemental Figure 6. The mediation effect of obese indices on the association between outdoor ALAN exposure (per-quartile increment) and insulin.

Abbreviations: *β*, correlation coefficient; *CI*, confidence interval; ALAN, artificial light at night; WC, waist circumference; BMI, body mass index; WHtR, waist-to-height ratio; WHR, waist-to-hip ratio; VFI, visceral fat index; BFP, body fat percentage.

Adjusted for age, gender, education status, marital status, per capita monthly income, smoking and drinking status, more vegetable and fruit intake, high-fat diet, physical activity, and family history of diabetes.

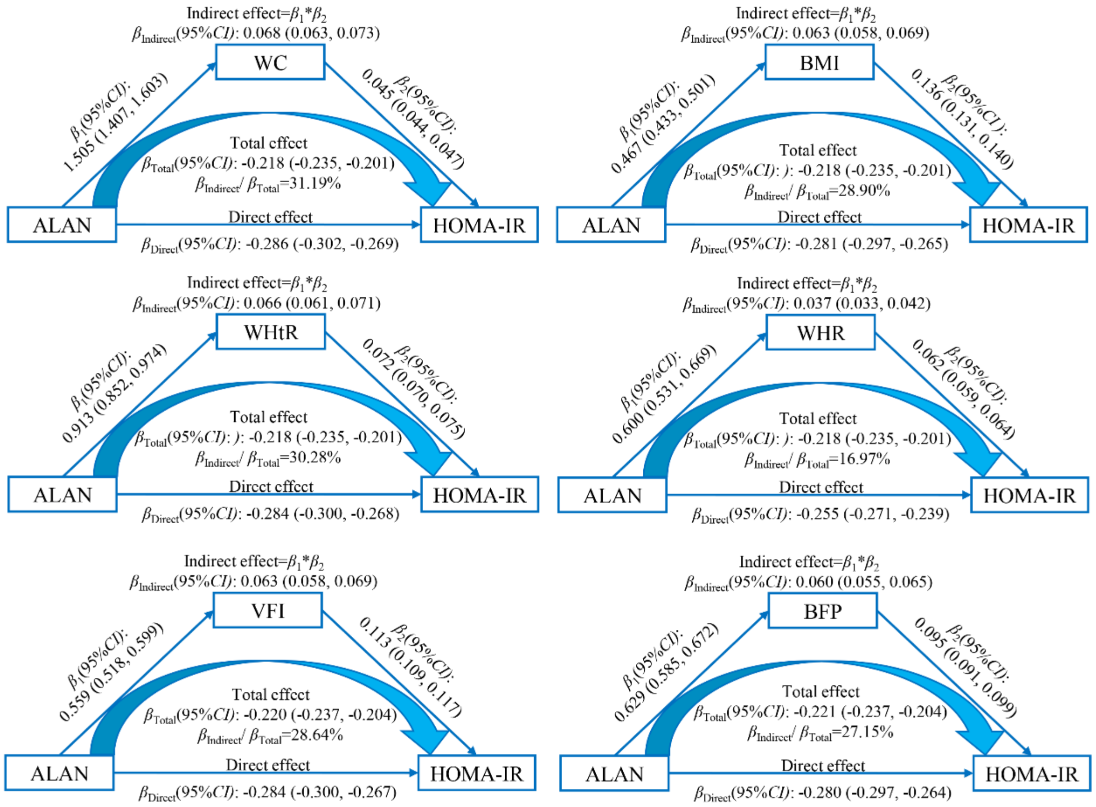

Supplemental Figure 7. The mediation effect of obese indices on the association between outdoor ALAN exposure (per-quartile increment) and HOMA-IR.

Abbreviations: β, correlation coefficient; *CI*, confidence interval; ALAN, artificial light at night; HOMA-IR: insulin resistance index; WC, waist circumference; BMI, body mass index; WHtR, waist-to-height ratio; WHR, waist-to-hip ratio; VFI, visceral fat index; BFP, body fat percentage.

Adjusted for age, gender, education status, marital status, per capita monthly income, smoking and drinking status, more vegetable and fruit intake, high-fat diet, physical activity, and family history of diabetes.

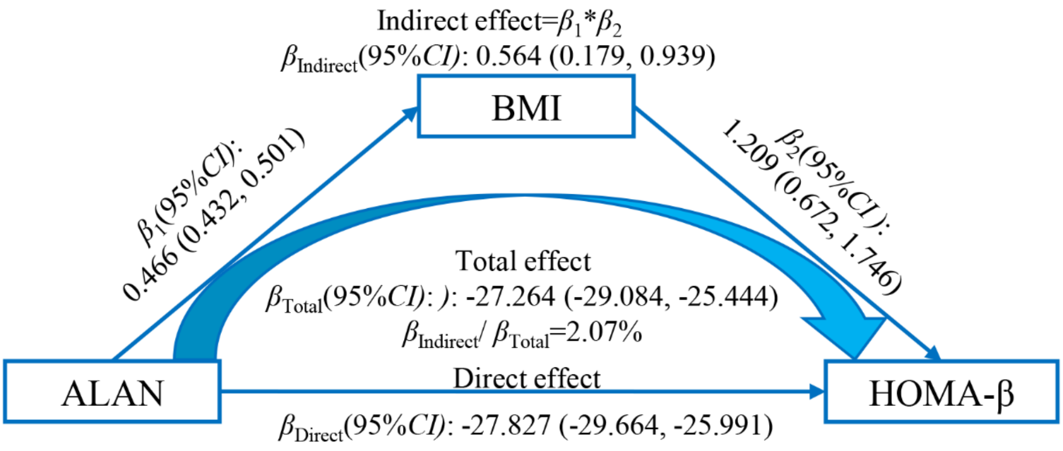

Supplemental Figure 8. The mediation effect of BMI on the association between outdoor ALAN exposure (per-quartile increment) and HOMA-β.

Abbreviations: *β*, correlation coefficient; *CI*, confidence interval; ALAN, artificial light at night; HOMA-β: β-cell function index; BMI, body mass index.

Adjusted for age, gender, education status, marital status, per capita monthly income, smoking and drinking status, more vegetable and fruit intake, high-fat diet, physical activity, and family history of diabetes.

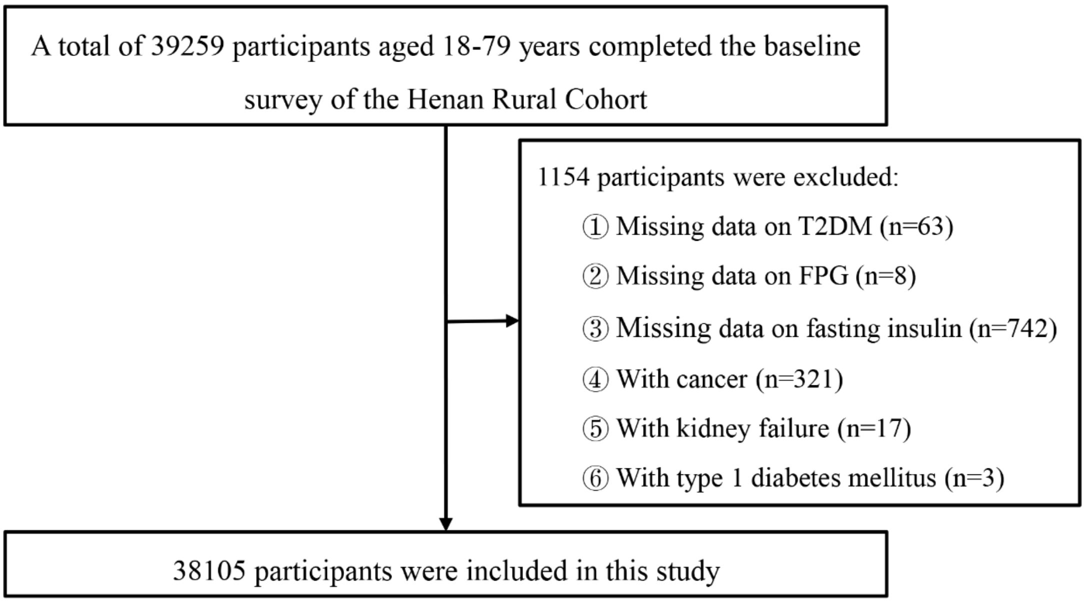

Supplemental Figure 9. The flowchart of participant selection

Abbreviations: T2DM: type 2 diabetes mellitus; FPG, fasting plasma glucose.

Supplemental Table 1. The associations of outdoor ALAN exposure with T2DM and glucose metabolism indexes further adjusting for the air pollution and green space based on model 3

ALAN	Model 10	Model 11	Model 12	Model 13	Model 14	Model 15	Model 16	Model 17
**T2DM (*OR*, 95%*CI*)**								
Q1 (≤0.21 nW/cm^2/sr)	1.00	1.00	1.00	1.00	1.00	1.00	1.00	1.00
Q2 (0.21-0.57 nW/cm^2/sr)	1.49 (1.33, 1.67)	1.47 (1.31, 1.65)	1.46 (1.31, 1.64)	1.45 (1.30, 1.63)	1.45 (1.30, 1.63)	1.45 (1.29, 1.62)	1.46 (1.30, 1.63)	1.45 (1.29, 1.62)
Q3 (0.57-1.83 nW/cm^2/sr)	2.14 (1.90, 2.40)	2.09 (1.86, 2.35)	1.94 (1.71, 2.19)	1.93 (1.70, 2.18)	1.75 (1.53, 2.01)	1.79 (1.56, 2.05)	1.87 (1.62, 2.16)	1.90 (1.64, 2.19)
Q4 (>1.83 nW/cm^2/sr)	1.80 (1.53, 2.12)	1.67 (1.42, 1.97)	1.69 (1.44, 2.00)	1.59 (1.35, 1.87)	1.58 (1.34, 1.87)	1.49 (1.26, 1.77)	1.67 (1.41, 1.98)	1.58 (1.33, 1.87)
P for trend	<0.001	<0.001	<0.001	<0.001	<0.001	<0.001	<0.001	<0.001
Per-quartile increase	1.31 (1.24, 1.37)	1.28 (1.21, 1.34)	1.24 (1.18, 1.31)	1.22 (1.16, 1.29)	1.20 (1.13, 1.26)	1.17 (1.11, 1.24)	1.21 (1.14, 1.28)	1.19 (1.12, 1.26)
Continuous	1.03 (1.01, 1.06)	1.02 (1.00, 1.05)	1.03 (1.00, 1.05)	1.02 (0.99, 1.05)	1.02 (1.00, 1.05)	1.01 (0.98, 1.04)	1.03 (1.00, 1.05)	1.02 (0.99, 1.04)
**FPG (*β*, 95%*CI*)**								
Q1 (≤0.21 nW/cm^2/sr)	1.00	1.00	1.00	1.00	1.00	1.00	1.00	1.00
Q2 (0.21-0.57 nW/cm^2/sr)	0.21 (0.17, 0.25)	0.21 (0.16, 0.25)	0.21 (0.17, 0.25)	0.21 (0.16, 0.25)	0.21 (0.17, 0.25)	0.21 (0.16, 0.25)	0.21 (0.17, 0.25)	0.20 (0.16, 0.25)
Q3 (0.57-1.83 nW/cm^2/sr)	0.53 (0.48, 0.57)	0.52 (0.47, 0.56)	0.50 (0.45, 0.55)	0.50 (0.45, 0.54)	0.47 (0.41, 0.52)	0.48 (0.43, 0.53)	0.49 (0.43, 0.55)	0.50 (0.44, 0.56)
Q4 (>1.83 nW/cm^2/sr)	0.41 (0.35, 0.48)	0.37 (0.31, 0.44)	0.39 (0.33, 0.46)	0.36 (0.29, 0.43)	0.37 (0.30, 0.44)	0.34 (0.27, 0.41)	0.39 (0.32, 0.46)	0.36 (0.29, 0.43)
P for trend	<0.001	<0.001	<0.001	<0.001	<0.001	<0.001	<0.001	<0.001
Per-quartile increase	0.20 (0.18, 0.22)	0.19 (0.17, 0.21)	0.18 (0.15, 0.20)	0.17 (0.15, 0.19)	0.16 (0.13, 0.18)	0.15 (0.13, 0.18)	0.16 (0.13, 0.18)	0.15 (0.13, 0.18)
Continuous	0.03 (0.02, 0.04)	0.02 (0.01, 0.04)	0.03 (0.02, 0.04)	0.02 (0.01, 0.03)	0.02 (0.01, 0.03)	0.02 (0.01, 0.03)	0.02 (0.01, 0.04)	0.02 (0.01, 0.03)
**Insulin (*β*, 95%*CI*)**								
Q1 (≤0.21 nW/cm^2/sr)	1.00	1.00	1.00	1.00	1.00	1.00	1.00	1.00
Q2 (0.21-0.57 nW/cm^2/sr)	-0.48 (-0.62, -0.34)	-0.61 (-0.76, -0.46)	-0.33 (-0.47, -0.18)	-0.45 (-0.60, -0.31)	-0.34 (-0.49, -0.20)	-0.45 (-0.60, -0.31)	-0.31 (-0.45, -0.17)	-0.42 (-0.56, -0.27)
Q3 (0.57-1.83 nW/cm^2/sr)	-2.77 (-2.92, -2.61)	-2.91 (-3.07, -2.75)	-2.38 (-2.55, -2.21)	-2.46 (-2.63, -2.29)	-2.27 (-2.46, -2.08)	-2.29 (-2.48, -2.10)	-2.19 (-2.39, -1.98)	-2.17 (-2.37, -1.97)
Q4 (>1.83 nW/cm^2/sr)	-2.37 (-2.60, -2.14)	-2.86 (-3.09, -2.63)	-2.27 (-2.50, -2.04)	-2.69 (-2.92, -2.45)	-2.20 (-2.44, -1.96)	-2.49 (-2.74, -2.25)	-2.18 (-2.43, -1.94)	-2.49 (-2.73, -2.24)
P for trend	<0.001	<0.001	<0.001	<0.001	<0.001	<0.001	<0.001	<0.001
Per-quartile increase	-1.08 (-1.15, -1.01)	-1.22 (-1.29, -1.15)	-0.92 (-1.00, -0.85)	-1.04 (-1.12, -0.97)	-0.82 (-0.90, -0.74)	-0.91 (-0.99, -0.83)	-0.77 (-0.85, -0.69)	-0.89 (-0.95, -0.79)
Continuous	-0.12 (-0.16, -0.08)	-0.18 (-0.22, -0.14)	-0.12 (-0.16, -0.08)	-0.19 (-0.23, -0.15)	-0.08 (-0.12, -0.04)	-0.13 (-0.17, -0.09)	-0.09 (-0.13, -0.05)	-0.15 (-0.19, -0.11)
**HOMA-IR (*β*, 95%*CI*)**								
Q1 (≤0.21 nW/cm^2/sr)	1.00	1.00	1.00	1.00	1.00	1.00	1.00	1.00
Q2 (0.21-0.57 nW/cm^2/sr)	0.02 (-0.03, 0.06)	-0.02 (-0.07, 0.03)	0.05 (0.00, 0.10)	0.02 (-0.03, 0.07)	0.05 (0.00, 0.10)	0.02 (-0.03, 0.07)	0.06 (0.01, 0.10)	0.03 (-0.02, 0.08)
Q3 (0.57-1.83 nW/cm^2/sr)	-0.41 (-0.47, -0.36)	-0.45 (-0.51, -0.40)	-0.33 (-0.39, -0.27)	-0.35 (-0.41, -0.30)	-0.31 (-0.38, -0.25)	-0.31 (-0.38, -0.25)	-0.28 (-0.35, -0.21)	-0.27 (-0.34, -0.21)

Q4 (＞1.83 nW/cm²/sr)	-0.37 (-0.44, -0.29)	-0.51 (-0.59, -0.43)	-0.35 (-0.43, -0.27)	-0.47 (-0.55, -0.39)	-0.34 (-0.42, -0.26)	-0.43 (-0.51, -0.35)	-0.33 (-0.41, -0.25)	-0.42 (-0.50, -0.34)
P for trend	<0.001	<0.001	<0.001	<0.001	<0.001	<0.001	<0.001	<0.001
Per-quartile increase	-0.17 (-0.19, -0.14)	-0.21 (-0.23, -0.18)	-0.14 (-0.16, -0.11)	-0.17 (-0.19, -0.15)	-0.12 (-0.14, -0.09)	-0.14 (-0.17, -0.12)	-0.11 (-0.13, -0.08)	-0.13 (-016, -0.11)
Continuous	-0.02 (-0.03, -0.00)	-0.04 (-0.05, -0.02)	-0.02 (-0.03, -0.01)	-0.04 (-0.05, -0.03)	-0.01 (-0.03, 0.00)	-0.03 (-0.04, -0.01)	-0.01 (-0.03, -0.00)	-0.03 (-0.04, -0.02)
**HOMA-β (*β*, 95%*CI*)**								
Q1 (≤0.21 nW/cm²/sr)	1.00	1.00	1.00	1.00	1.00	1.00	1.00	1.00
Q2 (0.21-0.57 nW/cm²/sr)	-20.90 (-26.17, -15.63)	-21.83 (-27.19, -16.47)	-19.16 (-24.42, -13.90)	-20.13 (-25.47, -14.80)	-19.26 (-24.53, -14.00)	-20.16 (-25.50, -14.83)	-18.79 (-24.05, -13.52)	-19.69 (-25.03, -14.36)
Q3 (0.57-1.83 nW/cm²/sr)	-74.08 (-79.84, -68.31)	-75.06 (-80.88, -69.23)	-69.16 (-75.38, -62.94)	-69.75 (-76.00, -63.50)	-66.63 (-73.53, -59.73)	-67.16 (-74.04, -60.29)	-64.63 (-71.94, -57.33)	-64.82 (-72.10, -57.53)
Q4 (＞1.83 nW/cm²/sr)	-62.83 (-71.18, -54.47)	-65.94 (-74.45, -57.43)	-61.24 (-69.69, -52.79)	-63.81 (-72.41, -55.21)	-59.61 (-68.33, -50.89)	-61.12 (-70.02, -52.22)	-58.69 (-67.52, -49.86)	-60.45 (-69.40, -51.50)
P for trend	<0.001	<0.001	<0.001	<0.001	<0.001	<0.001	<0.001	<0.001
Per-quartile increase	-28.57 (-31.09, -26.05)	-29.70 (-32.27, -27.14)	-25.80 (-28.43, -23.16)	-26.81 (-29.48, -24.14)	-23.27 (-26.06, -20.47)	-24.15 (-27.00, -21.30)	-21.95 (-24.82, -19.09)	-22.81 (-25.71, -19.91)
Continuous	-3.88 (-5.26, -2.50)	-4.10 (-5.52, -2.68)	-3.66 (-5.04, -2.29)	-4.05 (-5.46, -2.63)	-2.77 (-4.15, -1.39)	-2.91 (-4.33, -1.49)	-2.87 (-4.25, -1.49)	-3.21 (-4.63, -1.80)

Abbreviations: *OR*, odds ratio; *β*, correlation coefficient; *CI*, confidence interval; ALAN, artificial light at night; T2DM: type 2 diabetes mellitus; FPG, fasting plasma glucose; HOMA-IR: insulin resistance index; HOMA-β: β-cell function index; PM_1, particulate matter with aerodynamic diameters ≤1.0 μm; $PM_{2.5}$, particulate matter with aerodynamic diameters ≤2.5 μm; PM_{10}, particulate matter with aerodynamic diameters ≤10 μm; NO_2, nitrogen dioxide; EVI, Enhanced Vegetation Index; NDVI, Normalized Difference Vegetation Index.

Model 10 was additionally adjusted for PM_1 and EVI based on Model 3. Model 11 was additionally adjusted for PM_1 and NDVI based on Model 3. Model 12 was additionally adjusted for $PM_{2.5}$ and EVI based on Model 3. Model 13 was additionally adjusted for $PM_{2.5}$ and NDVI based on Model 3. Model 14 was additionally adjusted for PM_{10} and EVI based on Model 3. Model 15 was additionally adjusted for PM_{10} and NDVI based on Model 3. Model 16 was additionally adjusted for NO_2 and EVI based on Model 3. Model 17 was additionally adjusted for NO_2 and NDVI based on Model 3.

Supplementary Table 2. *P* values for the overall association test and the non-linear association test of outdoor ALAN exposure with T2DM and glucose metabolism indexes in the restricted cubic spline based on model 3*

ALAN	T2DM	FPG	Insulin	HOMA-IR	HOMA-*β*
χ^2 for the overall association test	188.86	570.47	3207.97	951.08	1201.79
P for the overall association test	<0.0001	<0.0001	<0.0001	<0.0001	<0.0001
χ^2 for the non-linear association test	148.99	479.41	2528.92	731.40	943.92
P for the non-linear association test	<0.0001	<0.0001	<0.0001	<0.0001	<0.0001

Abbreviations: ALAN, artificial light at night; T2DM: type 2 diabetes mellitus; FPG, fasting plasma glucose; HOMA-IR: insulin resistance index; HOMA-β: β-cell function index.

*Adjusted for age, sex, education level, per capita monthly income, smoking and drinking status, more vegetable and fruit intake, high-fat diet, physical activity, and family history of diabetes.

Supplemental Table 3. The mediation effects of obese indices on the associations of outdoor ALAN exposure (per-quartile increment) with T2DM and glucose metabolism indexes

Outcome	Mediator	Total effects<br>*β* (95% *CI*)	Indirect effects<br>*β* (95% *CI*)	Direct effects<br>*β* (95% *CI*)	ALAN-Obese indices<br>*β* (95% *CI*)	Obese indices-Outcome<br>*β* (95% *CI*)	Indirect/total effects (%)
T2DM							
	WC	0.206 (0.172, 0.241)	0.075 (0.068, 0.082)	0.130 (0.095, 0.165)	1.505 (1.407, 1.603)	0.050 (0.047, 0.054)	36.41
	BMI	0.207 (0.173, 0.242)	0.052 (0.046, 0.058)	0.156 (0.121, 0.192)	0.467 (0.433, 0.501)	0.112 (0.102, 0.121)	25.14
	WHtR	0.206 (0.172, 0.241)	0.070 (0.063, 0.078)	0.134 (0.099, 0.169)	0.913 (0.852, 0.974)	0.077 (0.071, 0.083)	33.98
	WHR	0.206 (0.172, 0.241)	0.046 (0.040, 0.053)	0.158 (0.123, 0.193)	0.601 (0.531, 0.670)	0.077 (0.072, 0.082)	22.33
	VFI	0.205 (0.171, 0.240)	0.050 (0.045, 0.056)	0.155 (0.119, 0.190)	0.559 (0.518, 0.599)	0.090 (0.082, 0.098)	24.39
	BFP	0.205 (0.170, 0.240)	0.053 (0.047, 0.060)	0.155 (0.120, 0.191)	0.629 (0.585, 0.672)	0.084 (0.076, 0.093)	25.85
FPG							
	WC	0.143 (0.129, 0.157)	0.035 (0.032, 0.038)	0.108 (0.094, 0.122)	1.505 (1.407, 1.603)	0.023 (0.022, 0.025)	24.48
	BMI	0.143 (0.129, 0.157)	0.025 (0.022, 0.027)	0.119 (0.104, 0.133)	0.467 (0.433, 0.501)	0.053 (0.049, 0.057)	17.48
	WHtR	0.143 (0.129, 0.157)	0.033 (0.030, 0.037)	0.110 (0.096, 0.124)	0.913 (0.852, 0.974)	0.036 (0.034, 0.039)	23.08
	WHR	0.143 (0.129, 0.157)	0.022 (0.020, 0.025)	0.121 (0.107, 0.135)	0.601 (0.531, 0.670)	0.037 (0.035, 0.039)	15.38
	VFI	0.142 (0.127, 0.156)	0.026 (0.023, 0.028)	0.116 (0.102, 0.130)	0.559 (0.518, 0.599)	0.046 (0.042, 0.049)	18.31
	BFP	0.141 (0.127, 0.156)	0.025 (0.023, 0.028)	0.116 (0.102, 0.130)	0.629 (0.585, 0.672)	0.040 (0.037, 0.044)	17.73
Insulin							
	WC	-1.197 (-1.247, -1.147)	0.197 (0.183, 0.213)	-1.394 (-1.443, -1.345)	1.505 (1.407, 1.603)	0.131 (0.126, 0.136)	16.46
	BMI	-1.197 (-1.247, -1.146)	0.198 (0.182, 0.216)	-1.394 (-1.443, -1.346)	0.467 (0.433, 0.501)	0.424 (0.410, 0.438)	16.54
	WHtR	-1.197 (-1.248, -1.147)	0.193 (0.178, 0.209)	-1.390 (-1.439, -1.341)	0.913 (0.852, 0.974)	0.211 (0.203, 0.219)	16.12
	WHR	-1.198 (-1.248, -1.147)	0.101 (0.088, 0.113)	-1.299 (-1.348, -1.250)	0.601 (0.531, 0.670)	0.169 (0.162, 0.176)	8.43
	VFI	-1.201 (-1.251, -1.151)	0.196 (0.181, 0.214)	-1.397 (-1.446, -1.349)	0.559 (0.518, 0.599)	0.351 (0.339, 0.363)	16.32
	BFP	-1.201 (-1.251, -1.151)	0.186 (0.172, 0.201)	-1.388 (-1.437, -1.339)	0.629 (0.585, 0.672)	0.296 (0.285, 0.308)	15.49
HOMA-IR							
	WC	-0.218 (-0.235, -0.201)	0.068 (0.063, 0.073)	-0.286 (-0.302, -0.269)	1.505 (1.406, 1.603)	0.045 (0.044, 0.047)	31.19

	BMI	-0.218 (-0.235, -0.201)	0.063 (0.058, 0.069)	-0.281 (-0.297, -0.265)	0.467 (0.433, 0.501)	0.136 (0.131, 0.140)	28.90
	WHtR	-0.218 (-0.235, -0.201)	0.066 (0.061, 0.071)	-0.284 (-0.300, -0.268)	0.913 (0.852, 0.974)	0.072 (0.070, 0.075)	30.28
	WHR	-0.218 (-0.235, -0.201)	0.037 (0.033, 0.042)	-0.255 (-0.271, -0.239)	0.600 (0.531, 0.669)	0.062 (0.059, 0.064)	16.97
	VFI	-0.220 (-0.237, -0.204)	0.063 (0.058, 0.069)	-0.284 (-0.300, -0.267)	0.559 (0.518, 0.599)	0.113 (0.109, 0.117)	28.64
	BFP	-0.221 (-0.237, -0.204)	0.060 (0.055, 0.065)	-0.280 (-0.297, -0.264)	0.629 (0.585, 0.672)	0.095 (0.091, 0.099)	27.15
HOMA-β							
	WC	-27.312 (-29.130, -25.494)	0.248 (-0.202, 0.642)	-27.560 (-29.400, -25.721)	1.504 (1.406, 1.602)	0.165 (-0.209, 0.351)	/
	BMI	-27.264 (-29.084, -25.444)	0.564 (0.179, 0.939)	-27.827 (-29.664, -25.991)	0.466 (0.432, 0.501)	1.209 (0.672, 1.746)	2.07
	WHtR	-27.314 (-29.132, -25.495)	0.315 (-0.112, 0.677)	-27.629 (-29.468, -25.790)	0.913 (0.852, 0.974)	0.345 (0.045, 0.646)	/
	WHR	-27.322 (-29.140, -25.503)	0.064 (-0.189, 0.264)	-27.385 (-29.211, -25.560)	0.600 (0.531, 0.669)	0.106 (-0.159, 0.370)	/
	VFI	-27.304 (-29.132, -25.475)	0.446 (-0.030, 0.796)	-27.750 (-29.596, -25.904)	0.558 (0.518, 0.599)	0.800 (0.344, 1.255)	/
	BFP	-27.300 (-29.128, -25.472)	0.483 (-0.009, 0.846)	-27.783 (-29.630, -25.935)	0.628 (0.585, 0.671)	0.768 (0.341, 1.195)	/

Abbreviations: *β*, correlation coefficient; *CI*, confidence interval; ALAN, artificial light at night; T2DM: type 2 diabetes mellitus; FPG, fasting plasma glucose; HOMA-IR: insulin resistance index; HOMA-β: β-cell function index; WC, waist circumference; BMI, body mass index; WHtR, waist-to-height ratio; WHR, waist-to-hip ratio; VFI, visceral fat index; BFP, body fat percentage.

Adjusted for age and gender, education status, marital status, per capita monthly income, smoking and drinking status, more vegetable and fruit intake, high-fat diet, physical activity, and family history of diabetes.
